# Olfactory ensheathing cell-derived extracellular vesicles protect the degenerated retinal pigment epithelium by delivering phagocytosis-related proteins

**DOI:** 10.20517/evcna.2026.50

**Published:** 2026-07-29

**Authors:** Siyu Chen, Xi Lin, Zhe Cha, Hui Gao, Jing Zhou, Shujia Huo, Cao Yang, Jiahui Kang, Lingyue Mo, Lijuan Yan, Yijian Li, Xinying Hu, Luodan A, Xiaotang Fan, Jing Xie, Haiwei Xu

**Affiliations:** ^1^Department of Ophthalmology, The First Affiliated Hospital of Army Medical University (Southwest Hospital), Chongqing 400038, China.; ^2^Chongqing Key Laboratory of Visual Injury and Regeneration, Chongqing 400038, China.; ^3^Shigatse Branch of Xinqiao Hospital, 953th Hospital, Army Medical University (Third Military Medical University), Shigatse 857000, Xizang, China.; ^4^Department of Ophthalmology, Jiangyin People’s Hospital, Medical School of Southeast University, Jiangyin 214432, Jiangsu, China.; ^5^Department of Military Cognitive Psychology, School of Psychology, Third Military Medical University (Army Medical University), Chongqing 400038, China.; ^#^These authors contributed equally to this work.

**Keywords:** Olfactory ensheathing cells, small extracellular vesicles, retinal pigment epithelium, retinitis pigmentosa, MerTK

## Abstract

**Aim:** Olfactory ensheathing cells (OECs) are commonly utilized in cell-based therapies for neurodegenerative diseases and central nervous system injuries, due to their unique properties of suppressing gliosis and providing neuroprotection. Small extracellular vesicles (sEVs) have emerged as promising therapeutic agents. This study aimed to investigate OEC-sEVs’ effect on retinal degeneration.

**Methods:** Proteomics analysis identified differentially expressed proteins between OEC-sEVs and OECs. We conducted subretinal injection of OEC-sEVs and assessed their protective mechanisms in degenerative retina. Real-time quantitative PCR and Western blot were used to detect the expression of phagocytosis-related molecules in retinal pigment epithelium (RPE) cells.

**Results:** Proteomics analysis revealed that proteins enriched in OEC-sEVs were associated with phagocytosis. Following injection into the subretinal spaces of Royal College of Surgeons (RCS) rats, OEC-sEVs were internalized by RPE cells, leading to the transient upregulation of tyrosine-protein kinase Mer (MerTK), activation of the downstream phosphoinositide 3-kinase/Akt signaling pathway, and restoration of the phagocytic activity of RPE cells towards the photoreceptor outer segments. This delayed photoreceptor degeneration, thereby improving retinal function in RCS rats. In the ARPE-19 cell line treated with the specific MerTK inhibitor UNC2881, enhanced phagocytosis of fluorescent particles and porcine photoreceptor outer segments was observed following OEC-sEV internalization.

**Conclusion:** OEC-sEVs were abundant in proteins associated with phagocytosis, which may partially compensate for the phagocytic deficit of the RPE cells observed in a retinitis pigmentosa rat model via the MerTK-related signaling pathway.

## INTRODUCTION

Retinitis pigmentosa (RP), characterized by the loss of retinal pigment epithelium (RPE) cells and photoreceptors, has emerged as a leading cause of blindness worldwide^[1,2]^. RPE cell dysfunction is a common feature of retinal degenerative diseases. RPE cells are located underneath the photoreceptors and play a vital role in pinching off the distal ends of the porcine photoreceptor outer segments (POS), providing nutrients for the photoreceptors and maintaining the visual cycle^[3,4]^. In vertebrates, photons are projected onto the POS, guaranteeing that sensitive light detection is essential for image vision^[5]^. Unfortunately, this procedure can cause oxidative damage to the POS, thereby deteriorating visual function. Over time, vertebrates erect safeguards against light damage to prevent cumulative damage to photoreceptors, a process that boils down to “new for old” and is mediated by RPE cells^[6,7]^. Daily clearance and phagocytosis of POS by RPE cells have been verified to ensure retinal homeostasis and longevity of photoreceptors^[5,8]^. Accordingly, degeneration of the RPE could lead to the loss of photoreceptors and, ultimately, retinal degeneration. Currently, no effective therapy is available in clinical practice^[9]^. Various experimental therapeutic approaches have been explored for RP, including stem cell therapy, gene therapy, and retinal prosthesis implants^[10,11]^. Cell-based therapy is a potential treatment for RP and has been tested in clinical trials.

As an attractive approach, cell-based therapy has utilized multiple cell sources, all of which demonstrate therapeutic potential^[12,13]^. In particular, olfactory ensheathing cells (OECs) have emerged as a promising cell source, exhibiting immense potential to ameliorate RP^[14]^. OECs are a specialized type of glial cell that shares numerous properties with both astrocytes and Schwann cells^[15]^. Substantial evidence indicates that OECs possess superior phagocytic properties, anti-inflammatory activity, and neuroprotective effects^[16-20]^. Notably, transplantation of OECs/olfactory nerve fibroblasts (ONFs) into the damaged central nervous system (CNS) can stimulate axonal regeneration and restore visual function^[15,21,22]^. Moreover, OEC transplantation can inhibit microglial activation, reduce inflammation in the retina and spinal cord, and promote neurogenesis and remyelination in spinal cord injuries^[23]^. While OECs exhibit multiple therapeutic properties, their phagocytic capacity appears particularly relevant for treating phagocytosis-deficient disorders. Previous studies have demonstrated OECs’ ability to continuously phagocytose neuronal debris and bacteria^[17,24]^. However, whether OECs directly restore impaired RPE cells in RP models remains unclear. Despite this knowledge gap, OEC transplantation has shown promising therapeutic outcomes for RP treatment.

Small extracellular vesicles (sEVs), measuring 30-200 nm, are released by cells. sEVs can mediate intercellular communication by transferring cargo, including proteins, lipids, microRNAs, and mRNA^[25,26]^. In recent years, emerging evidence has proven that sEVs, derived from diverse cell types, particularly stem cells, represent a promising strategy for treating ophthalmic diseases. Compared to their parent cells, sEVs exhibit superior biocompatibility and safety, lower immunogenicity, greater ease of passage across various biological barriers, and improved storage stability^[27]^. These properties position sEVs as ideal cell-free candidates for tissue repair and regeneration^[28]^.

Among sEVs from commonly studied cell sources, including mesenchymal stem cells (MSCs), neural progenitor cells (NPCs), retinal progenitor cells (RPCs), human embryonic stem cells (hESCs), RPE cells, and OECs^[29-32]^, OEC-derived sEVs (OEC-sEVs) are gaining prominence in suppressing inflammation, promoting axonal growth, and enhancing nerve regeneration^[33-35]^. Notably, Tu *et al*., performed the first miRNA profiling of human OEC-sEVs and validated their neuroprotective effects on NPCs^[36]^. Nevertheless, the ability of OEC-sEVs to enhance phagocytic activity in phagocytosis-deficient cells remains unexplored. We hypothesized that OEC-sEVs exhibit strong therapeutic efficacy in the retinas of Royal College of Surgeons (RCS) rats by enhancing RPE cell phagocytosis.

Many studies have investigated the receptors involved in the phagocytosis of RPE cells. Mannose receptors were the first receptors discovered to be involved in phagocytosis, followed by muscarinic receptors, CD36, αvβ5 integrin, and tyrosine-protein kinase Mer (MerTK) receptors^[5]^. Various receptors exhibit different functions. Herein, CD36, αvβ5 integrin, and MerTK receptors play important roles in the uptake of POS, hence garnering considerable attention in the field. It has been verified that αvβ5 integrin can participate in recognition and surface binding, while MerTK and CD36 receptors are required in the endocytosis process of POS^[37-40]^. Several studies have shown that MerTK, a member of the Tyro3/Axl/Mer (TAM) RTK subfamily, is an indispensable transmembrane protein that regulates POS phagocytosis^[40-42]^.

RCS rats are a model for studying RP disease and carry a mutation in the *Mertk* gene^[43]^, which encodes the membrane receptor MerTK, expressed on phagocytes, including RPE cells, and enhances their engulfment. Accordingly, MerTK deficiency in RCS rats results in impairment of RPE phagocytosis and accumulation of outer segment (OS) debris in the subretinal space, ultimately leading to the subsequent loss of photoreceptors^[42,44]^. However, whether OEC-sEVs can compensate for the deficiency in phagocytosis by RPE cells in RCS rats remains unclear.

In the present study, we successfully harvested OEC-sEVs and demonstrated their internalization by RPE cells, thereby delaying photoreceptor degeneration and improving retinal function in RCS rats. Proteomic analysis revealed differentially expressed proteins (DEPs) between OEC-sEVs and OECs, confirming that the protein cargoes of OEC-sEVs were substantially related to phagocytosis compared with those in OECs, providing a rational basis for subsequent mechanistic investigations. Furthermore, we verified that OEC-sEVs delayed the progression of RP by partially compensating for the phagocytic deficit in RPE cells through a MerTK-related pathway, as demonstrated by both *in vivo* and *in vitro* experiments. Overall, we developed an innovative therapeutic paradigm for the application of optimized sEVs in RP.

## METHODS

### Animals

Long-Evans (LE) rats and RCS rats were provided by the Experimental Animal Center of Third Military University (Army Medical University)^[45]^. The animals were raised in a specific pathogen-free room at the Animal Care Center of Southwest Hospital, regardless of sex. RCS rats and LE rats were raised for 3 weeks for the experiments. The animals were randomly divided into experimental groups. A total of 55 RCS rats and 180 LE rats were used in our experiment. All procedures related to the animal experiments were conducted in accordance with the guidelines of the Association for Research in Vision and Ophthalmology (ARVO). All the experimental procedures were approved by the Laboratory Animal Welfare and Ethics Committee of the Third Military Medical University (Army Medical University, No. AMUWEC2020610).

### OEC culture

OECs were isolated from the olfactory bulbs of LE rats aged 8-12 weeks, following previously described protocols^[46,47]^. Each OEC batch was derived from the olfactory bulbs of 30 LE rats. The olfactory bulb tissue was dissociated with TrypLE (Thermo Fisher Scientific) and neutralized with DMEM/F-12 (GIBCO, c11330500BT) containing 10% fetal bovine serum (FBS). The cell suspension was centrifuged at 350 × *g* for 5 min, the supernatant was discarded, and the pellet was resuspended in DMEM/F-12 supplemented with 10% FBS and penicillin-streptomycin (Thermo Fisher Scientific). The cells were plated on an uncoated dish and incubated at 37 °C for 18 h to remove fibroblasts. The floating cells were then transferred to another dish for astrocyte removal and incubated for 36 h. Subsequently, OECs from the resulting cell suspension were seeded onto a laminin-pretreated (40 µg/mL, Life Technologies) 150-mm dish and cultured in DMEM/F-12 complete medium containing 10% FBS and 100 U/mL penicillin-streptomycin. The culture was further supplemented with 0.01 mg/L basic fibroblast growth factor (bFGF) (MCE, RAT HY-P7091) and 2 µM forskolin (Sigma, F6886) to support cell proliferation. OECs were maintained at 37 °C in a 5% CO_2_ atmosphere, with the medium replaced every 3 days. The conditioned medium was collected exclusively from OECs at passage 3. Upon reaching approximately 90% confluency, the FBS was replaced with 10% exosome-depleted FBS (VivaCell, C3801-0050). After 48 h of cell growth, the medium was collected for exosome isolation.

### ARPE-19 cell culture and UNC2881 treatment

ARPE-19 cells (RRID: CVCL_0145) were purchased from Procell Life Science & Technology Co., Ltd. (CL-0026; China). ARPE-19 cells were cultured in DMEM/Ham’s F-12 medium supplemented with 10% FBS and 100 U/mL penicillin in a CO_2_ incubator at 37 ℃. Cell identity was authenticated by short tandem repeat (STR) profiling, which confirmed that the cells were authentic ARPE-19 cells and verified the absence of cross-contamination with other human cell lines. Mycoplasma contamination was routinely monitored using Polymerase Chain Reaction (PCR)-based/colorimetric detection methods, which yielded negative results. For all the experiments described in this study, ARPE-19 cells were used within passages 6-10 to minimize phenotypic drift.

UNC2881 (MedChemExpress, HY-15798) is a commercially available, selective Mer kinase inhibitor that effectively inhibits Mer kinase phosphorylation at the steady state^[48,49]^. Once ARPE19 cells reached 80% confluency, they were treated with UNC2881 at 1, 3, 5, 10, 25, and 50 µM for 90 min. ARPE-19 cells were detached using 0.25% trypsin for subsequent experiments.

### *In vitro* phagocytosis assay of porcine POS

POS was isolated from fresh porcine eyes obtained from a slaughterhouse using a modified method described by Molday *et al*.^[50]^. The retinas were homogenized by shaking in 20% sucrose, 65 mM NaCl, 1 mM MgCl_2_, 10 mM glucose, and 5 mM taurine in 20 mM Tris-HCl (pH 7.4). Large pieces of debris were pelleted at 1,000 rpm for 5 min. The pellet was resuspended in the same buffer and centrifuged at 15,000 rpm for 2 min at 4 °C, then resuspended again and centrifuged at 15,000 rpm for 30 min at 4 °C. POS pellets were stored at -80 °C in 10 mM sodium phosphate (pH 7.2), 0.1 M NaCl, and 2.5% sucrose. Before use, POS were labeled with 1 mg/mL fluorescein isothiocyanate (FITC; Invitrogen) in 0.1 M sodium bicarbonate (pH 9.0) for 1 h at room temperature, washed, and resuspended in culture medium. RPE cells were plated in 24-well plates at 1 × 10^5^ cells/well. After cell attachment, 50 μL of a suspension containing 2 × 10^6^ fluorescent POS was added to each well, followed by incubation at 37 °C for 24 h. To quench external fluorescence, samples were treated with 0.2% trypan blue in PBS-CM for 10 min before fixation in 4% paraformaldehyde (PFA) in PBS for 20 min at 4 °C. RPE cells were labeled with 4,6-diamidino-2-phenylindole (DAPI), followed by incubation with goat antirabbit IgGFITC secondary antibody.

### sEV isolation

According to our previous report^[51]^, with some modifications, two frequently used methods for isolating sEVs have been applied. For *in vivo* experiments, sEVs were purified via ultracentrifugation. OECs were dissociated into single cells and plated in a flask for 48 h in 15 mL culture medium containing 10% exosome-free serum (VivaCell, C3801-0050) per 150 mm cell culture dish. The supernatant was then subjected to ultracentrifugation at 100,000 × *g* for 70 min at 4 °C to pellet crude sEVs. The pellet was resuspended in PBS, filtered through a 220-nm membrane, and ultracentrifuged again under the same conditions for further purification. For *in vitro* experiments, sEVs were isolated using the ExoQuick-TC (SBI, System Biosciences, EXOTC10A-1) according to the manufacturer’s protocol. Cell culture supernatant was centrifuged at 3,000 × *g* for 15 min to remove cells and debris. The clarified supernatant was mixed with ExoQuick-TC solution and incubated overnight at 4 °C. The mixture was then centrifuged at 1,500 × *g* for 30 min at 4 °C, yielding a beige or white sEV pellet. After careful aspiration of the supernatant, the pellet was washed by brief centrifugation (1,500 × *g*, 5 min) to remove residual reagent, and finally resuspended in 200 µL PBS for further applications. We have submitted all relevant data on sEV isolation, characterization, and analysis to the EV-TRACK knowledge base (EV-TRACK ID: EV260006)^[52]^.

### PKH26 staining

According to the manufacturer’s instructions, sEVs were stained with a final staining volume of 2 mL containing 2 µM PKH26 (Sigma, product code MINI26). The sEV pellet was resuspended in 1 mL of Diluent C to prepare a 2× sEV suspension, and then gently pipetted to ensure complete dispersion. Simultaneously, a 2× dye solution was prepared at 4 µM by combining 4 µL of the ethanolic PKH26 stock with 1 mL of Diluent C in a polypropylene centrifuge tube, followed by thorough mixing. The staining reaction was initiated by rapidly combining equal volumes (1 mL each) of the 2× sEV suspension and 2× dye solution. The mixture was then incubated for 5 min with periodic mixing. To terminate the staining process, 2 mL of 1% bovine serum albumin was added to PBS to a final volume of 70 mL. To remove the unbound dye and isolate the stained sEVs, the samples were subjected to ultracentrifugation at 100,000 × *g* for 70 min. Finally, the pelleted sEVs were resuspended in PBS and stored at 4 °C for further use.

### Nanoparticle tracking analysis

The size distribution of sEVs was analyzed using a ZetaView instrument (Particle Metrix, Germany) as previously reported^[53]^. A freshly prepared concentrated sEV sample (1 µL) was diluted in 1 mL of PBS and mixed by gentle pipetting for 10 s. The diluted sample was then introduced into the ZetaView instrument following the manufacturer’s standardized protocol. Measurements were performed under the following conditions: 11 distinct positions were analyzed with the sensitivity set to 80, shutter set to 100, and frame rate set to 30. The system was operated with autofocus alignment and a laser wavelength of 488 nm at 25 °C and pH 7.4. Finally, the captured dynamic images were processed using ZetaView Software (version 8.05.12 SP2) to determine the sEV concentration and size distribution.

### Western Blot

Retinal hemispheres containing the injection area were isolated from RCS rats at 2 and 4 weeks post-operation (PO). Protein extraction from retinal tissues and OECs was performed using a lysis buffer composed of RIPA and PMSF (at a 9:1 ratio). For protein extraction from OEC-sEVs isolated by ultracentrifugation, the resuspended sEV samples were mixed with sodium dodecyl sulfate-polyacrylamide gel (SDS-PAGE) sample loading buffer (Beyotime, P0015) and heated at 100 °C for 10 min. Proteins (30 µg per lane) were separated on 10% SDS-PAGE electrophoresis and subsequently transferred onto polyvinylidene fluoride (PVDF) membranes. The membranes were blocked with 5% bovine serum albumin (BSA) for 1 h at ambient temperature, then incubated with specific primary antibodies at 4 °C overnight. The primary antibodies used included: rabbit anti-CD63 (Invitrogen, MA5-31980; 1:200), rabbit anti-CD9 (Invitrogen, MA5-31980; 1:200), TSG101 (Bioworld, BS6042; 1:1,000), mouse anti-GM130 (Boster, M05865-2; 1:1,000), rabbit anti-phosphoinositide 3-kinase (PI3K) (CST, 4257T, 1:200), rabbit anti-Phospho-PI3K (affbiotech, AF3242, 1:500), rabbit anti-MERTK (abcam, ab14921, 1:200), and mouse anti-β-actin (Beyotime, AF0003; 1:1,000). After washing, the membranes were incubated with horseradish peroxidase (HRP)-conjugated goat anti-mouse IgG (H + L) (Bioss, bs-0296G-HRP; 1:1,000) or HRP-conjugated goat anti-rabbit IgG (H + L) (Bioss, bs-0295G-HRP; 1:1,000) for 1 h at ambient temperature. Protein bands were visualized using Pierce^TM^ ECL Western Blot Substrate (Thermo, 32106) and imaged with a Bio-Rad detection system. Relative protein expression levels were normalized to β-actin and quantified using ImageJ software (version 1.53). For each biological replicate (*n* = 3 per group), three technical replicates were performed, and the average values were used for quantification.

### Subretinal injection

Subretinal injection was conducted in RCS rats (postnatal 3 weeks) with PKH26-labeled sEVs. The rats were anesthetized by intraperitoneal injection of a 1% sodium solution of pentobarbital solution (Thermo Fisher, Waltham, MA, USA) at a dose of 2.5 mL/kg. Oxybuprocaine hydrochloride was administered to anesthetize the ocular surface. A concentrated solution of OEC-sEVs (3.5 µL, containing 1.0 × 10^9^ particles/mL sEVs) was injected into the subretinal space of one eye of each RCS rat. The contralateral eye was injected with 3.5 µL of sterilized PBS (vehicle). Following the surgical procedure, the ocular surface was treated with tobramycin-dexamethasone eye ointment (Dian Bishu).

### Flash electroretinogram recording

Visual function was tested with a full-field flash ERG system (PuREC and LED Visual Stimulator LS-100/200; Mayo Corporation, Aichi, Japan). Corneal scotopic flash electroretinogram recordings (FERGs) from both eyes of the rats were performed 7, 14, and 28 days post-operation. Following a 12-h dark adaptation period, the animals were anesthetized with 1% pentobarbital sodium. Pupils were then dilated using topical administration of 1% tropicamide and 0.4% oxybuprocaine hydrochloride eye drops. Throughout the procedure, body temperature was maintained at 37 °C using a heating pad. Each cornea was connected to two active gold electrodes that served as the recording electrodes. The reference and ground electrodes were placed subcutaneously in the mid-frontal areas of the head and tail, respectively. Light stimuli elicited retinal responses at five different intensities: -2.5, -0.5, -0.02, 0.5, and 1 log (cd·s/m^2^). The a-wave and b-wave amplitudes were recorded and processed using the RETI-Port system. All procedures were performed in a dark room under a dim red safety light. The acquired data were analyzed using GraphPad Prism software (version 9.5.1.733). The a-wave amplitude indicates outer retinal function, whereas the b-wave amplitude reflects inner retinal signal transmission. Implicit times of both waves provide additional information on the kinetics of phototransduction and synaptic processing. Reduced amplitudes or prolonged implicit times suggest corresponding retinal dysfunction.

### Transmission electron microscopy

For transmission electron microscopy (TEM), OEC-sEVs were suspended in 50 µL PBS and transferred to formvar-coated copper grids, which were incubated for 20 min at ambient temperature. The samples were fixed in a solution of 2% PFA, 2% glutaraldehyde, and 0.05 M phosphate solution for 2 min. Following fixation, the grids were washed three times with distilled water and subsequently treated with 1% phosphotungstic acid (PTA) for 1 min. Excess liquid was removed using filter paper. The grids were dried overnight, observed, and analyzed using a transmission electron microscope (JEM-1400PLUS, JEOL, Japan).

### RPE whole-mount preparation and staining

Rats were anesthetized and perfused with 0.9% saline, after which the eyeballs were enucleated and fixed in 4% PFA for 1 h. The retinas were then carefully dissected under a microscope (Olympus, Tokyo, Japan) after removal of the cornea, iris, and lens. Each retina was radially incised into a clover-like pattern centered on the optic disc to prepare the whole-mount samples. The RPE whole mounts were subsequently treated with 10% hydrogen peroxide to bleach melanin at 55 °C for 1 h, followed by rinsing in PBS. Thereafter, the samples were permeabilized using a solution containing 3 g/L Triton X-100 at 37 °C for 12 h, and further blocked with 3% bovine serum albumin (BSA) and 3% FBS for another 12 h. Immunostaining was performed by incubating the whole mounts with 647-conjugated Phalloidin (Abcam, AB176759) and anti-Opsin antibody (Invitrogen, PA1-9517) at 4 °C for 12 h, followed by incubation with Alexa Fluor 488-conjugated goat anti-chicken secondary antibody (Invitrogen, A11039) at 4 °C overnight. Finally, the samples were mounted using ProLong^TM^ Diamond antifade mountant (Invitrogen, P36961) and imaged with a confocal microscope (Zeiss LSM880). Detailed information on the antibodies and reagents is provided in Supplementary Table 1.

### Immunofluorescence staining

For retinal tissue, OECs, and ARPE-19 preparations, immunofluorescence (IF) staining was performed as previously described^[51]^. Rats were euthanized via CO_2_ inhalation at 2 to 4 weeks post-operation, after which the eyeballs were enucleated. The resulting eye cups were fixed in 4% PFA for 1 h at 4 °C and subsequently dehydrated overnight in 30% glucose solution. Tissue samples were embedded in an optimal cutting temperature (OCT) compound and sectioned using a microtome (Leica, CM1900). Sections were mounted on glass slides, washed three times with PBS, and blocked with a solution containing 5% BSA and 0.3% Triton X-100 for 30 min at 37 °C. For immunolabeling, sections were incubated overnight with the following primary antibodies: arrestin (Sigma, ab15282), PKC-α (Abcam, ab32376), TUNEL (Roche, 12156792910), RPE65 (Santa Cruz, sc-390787), S100 (Boster, M009791), P75 (abcam, ab52987), and F-actin (Invitrogen, MA1-80729). The following day, after PBS washes, samples were incubated with species-appropriate secondary antibodies conjugated to Alexa Fluor 488, 568, or 647 (Invitrogen), along with DAPI for nuclear staining. Detailed information on antibody dilutions and sources is provided in Supplementary Table 1. Confocal z-stack images were acquired using a confocal microscope (Zeiss LSM880). Three-dimensional (3D) surface reconstruction was performed using Imaris software (version 10.2.0, Bitplane, Oxford Instruments).

### Analysis of relative fluorescence intensity

We utilized ImageJ to measure the average fluorescence intensity as described in a previous report^[54]^. The relative fluorescence intensity was quantified using ImageJ software. For each image, the mean gray value of the region of interest (ROI) was measured and subtracted from the background intensity of the adjacent unstained area. The intensity values were then normalized to the control group (set to 1.0) to obtain relative fluorescence units. To account for the strong background and inhomogeneous staining patterns, we selected multiple representative fields from each sample, avoiding areas with excessive background or artifacts, and the local background correction was applied by measuring adjacent non-specific regions. Consistent intensity thresholds were applied across all images to minimize variability. Five ROIs per sample were analyzed and averaged to reduce the impact of inhomogeneity.

### Analysis of outer nuclear layer (ONL)

The outer nuclear layer (ONL) of the retina was stained with DAPI and scanned under a confocal microscope (Zeiss LSM880). For ONL thickness quantification, the ROI was centered on the sEVs injection site. Three measurement points were selected within a 60 µm region to the left and three within a 60 µm region to the right of the injection site. The ONL thickness for each retinal sample was then calculated as the mean value of these six measurement points. Four retinas were included in each group for the analysis. ImageJ software was used to evaluate the ONL thickness in each group.

### β-hydroxybutyric acid assay

A microplate reader was preheated for 30 min and calibrated at 340 nm. All reagents in the β-hydroxybutyric acid (β-HB) content assay kit (RXWB0822-96) were thawed to room temperature, and samples and reagents were added sequentially into a 96-well plate according to the manufacturer’s instructions. The reagents were mixed thoroughly and incubated at 37 ℃ for a specified period, after which the absorbance was measured at 340 nm. Then the results were calculated with the following formula mentioned in the instructions.

### RNA isolation and quantitative real-time PCR

Following the manufacturer’s instructions, retinal tissue was lysed in TRIzol reagent (Takara, Japan, TCH021), and total RNA was extracted for quantitative real-time PCR (qRT-PCR). Afterward, the RNA was reverse-transcripted into cDNA using the PrimeScript RT reagent Kit with gDNA Eraser (TakaRa Bio, Japan, RR047A), with 2 µg RNA added into a 40 µL system. qRT-PCR was carried out using SYBR Premix Ex Taq (TakaRa Bio, Japan, RR082A), with 50 ng cDNA added into a 20 µL reaction. Then the expression levels of different genes were determined with 2^-ΔΔCT^ method, relative to the control gene (β-actin). The sequences of all the primers are shown in Supplementary Table 2.

### Flow cytometry

For cell preparation, the ARPE19 cells were digested and centrifuged. The cell pellet was then resuspended in the DMEM/F12 medium. The following experiments were conducted according to the manufacturer’s instructions. The pHrodo^TM^ Red *E. coli* BioParticles^TM^ conjugate for phagocytosis (Invitrogen, P35361) was resuspended in buffer and then sonicated for 10 min to ensure uniform dispersion of the fluorescent particles. After the cells had adhered, the culture medium was removed from each of the microplate wells by vacuum aspiration. The particle suspension was then added to a cell culture microplate at a 1:10 dilution. Afterward, the microplate was transferred to an incubator at 37 ℃ for 2 h, allowing phagocytosis and acidification to reach their maximum. All flow cytometry assays were performed using a BD FACS Calibur and BD FACS Aria II flow cytometer, and FlowJo software was used to analyze the data.

### Feret diameter measurement

The Feret diameter was measured using ImageJ software (version 1.53t, National Institutes of Health). RPE cells were manually delineated using a polygon selection tool to precisely outline individual cell boundaries based on phalloidin-labeled cortical actin. To ensure measurement accuracy, cells with overlapping boundaries, incomplete morphology, or abnormal spreading were excluded from the analysis. The Feret diameter data were exported to OriginPro software (version 2024, OriginLab Corporation) for statistical analysis and visualization. Non-linear Gaussian curve fitting was performed using a built-in Gaussian function.

### Proteomics analysis

Protein samples were prepared in triplicate for each experimental group. Each sample was homogenized in lysis buffer containing 7 M urea, 2% SDS, and a protease inhibitor cocktail, in a volume equivalent to that of the original sample. Homogenization was performed by sonication on ice using a sonicator (Qsonica, Q700) with a cycle of 2 s of sonication followed by 5 s of rest, repeated over 1 min. The lysate was then incubated on ice for 2 h, followed by centrifugation at 13,000 rpm for 20 min at 4 °C. The supernatant was collected, and the protein concentration was determined using a bicinchoninic acid (BCA) assay.

For enzymatic digestion, 50 µg of protein was aliquoted and subjected to reduction with 10 mM dithiothreitol (DTT) at 37 °C for 1 h, followed by alkylation with iodoacetamide (IAA) at a volumetric ratio of 1:5 (DTT: IAA) for 40 min at 37 °C. Proteins were precipitated by adding a five-fold volume of precipitation reagent, incubated for 1 h, and centrifuged at 13,000 rpm for 1 h at 4 °C. The resulting pellet was washed with 1 mL of 100% acetone and centrifuged again under the same conditions for 30 min. After air-drying for 10 min at room temperature, the pellet was digested with trypsin overnight at 37 °C.

After digestion, the peptides were desalted on a Monospin C18 column, dried under vacuum, and reconstituted in 0.1% formic acid. Approximately 1-2 µg of peptides were separated using an EASY-nLC 1200 system (Thermo Scientific) equipped with a C18 analytical column (1.9 µm, 75 µm × 20 cm) at a flow rate of 200 nL/min. Mass spectrometry analysis was performed on an Orbitrap Exploris 480 instrument coupled to a FAIMS Pro interface (Thermo Scientific) operating in the data-dependent acquisition mode. Full-scan mass spectrometry (MS) spectra were acquired at a resolution of 60,000 over the m/z range of 350-1,600 with higher-energy collisional dissociation (HCD) fragmentation at 30% collision energy. Data were processed using Proteome Discoverer software (version 2.4, Thermo Scientific).

Mass spectrometry proteomic data were deposited in the ProteomeXchange Consortium (https://proteomecentral.proteomexchange.org) via the iProX partner repository^[55,56]^ with the dataset identifier PXD072169.

### Bioinformatic analysis

Using Proteome Discoverer 2.4 software with the UniProt rat database as a reference, peptide and protein identification was performed under strict false discovery rate (FDR < 1%) control using specific enzyme digestion and modification parameters. Label-free relative protein quantification was conducted based on precursor ion intensity (Precursor Intensity) in the first-level mass spectrometry, followed by data normalization. Proteins exhibiting a fold change greater than 2 or less than -0.5, along with a *P*-value of less than 0.05 between the two groups, were identified as DEPs. We performed Gene Ontology (GO) enrichment analysis and Kyoto Encyclopedia of Genes and Genomes (KEGG) pathway enrichment analyses of the DEPs identified across groups. Protein-Protein Interaction (PPI) network construction and visualization were performed using Cytoscape (version 3.10.4, National Resource for Network Biology).

### Statistical analysis

All data are expressed as means ± standard deviation (SD), derived from at least four biological samples. GraphPad Prism 9.5.1.733 was used for the statistical analyses. Comparisons between two groups were performed using *t*-tests (and nonparametric alternatives), and comparisons among three groups were performed using one-way analysis ANOVA, followed by Tukey’s multiple-comparison test. Statistical significance was set at *P* < 0.05.

## RESULTS

### sEV isolation and identification

To assess the protective efficacy of the OEC-sEVs, we conducted both *in vivo* and *in vitro* experiments. OECs were isolated from the olfactory bulbs of LE rats, purified, and cultured. In *in vivo* studies, sEVs were extracted from the cell culture supernatant via ultracentrifugation and injected into the subretinal space of RCS rats. For the *in vitro* experiments, sEVs were isolated from the supernatant using an sEV extraction kit (ExoQuick-TC) and employed for further validation in ARPE-19 cells [Figure 1A]. We first examined the representative morphology of the OECs using phase-contrast microscopy. As shown in Figure 1B, the 7-day cultivation of OECs resulted in a cluster of cells with an elongated, spindle-shaped morphology. Immunocytochemistry analysis revealed high expression of S100 and P75, classic biomarkers of OECs, further confirming the OEC identity [Figure 1C and D]. For subsequent experiments, sEVs were isolated from these OECs using ultracentrifugation or ExoQuick-TC for *in vivo* and *in vitro* experiments, respectively. TEM images showed typical cup-shaped membrane vesicles with a homogeneous appearance, ranging from 30 to 200 nm in diameter [Figures 1E and F]. Besides, Western blot analysis confirmed the enrichment of sEV markers (CD63, CD9, and TSG101) in OEC-sEVs, whereas GM130, a cis-Golgi matrix protein absent in sEVs^[57]^, was undetectable [Figure 1G]. Nanoparticle tracking analysis (NTA) revealed a homogeneous population of sEV particles isolated by ultracentrifugation, with low dispersion [Figure 1H and I]. According to the particle size analysis results, the peak size of sEVs obtained by ultracentrifugation was 101-150 nm, and the mean size was 142.2 ± 65.24 nm [Figure 1J]. sEVs isolated via ExoQuick-TC showed lower purity but higher density than those isolated by ultracentrifugation [Figure 1K and L], and they had an average size of 134.86 ± 6.23 nm [Figure 1M].

**Figure 1:**
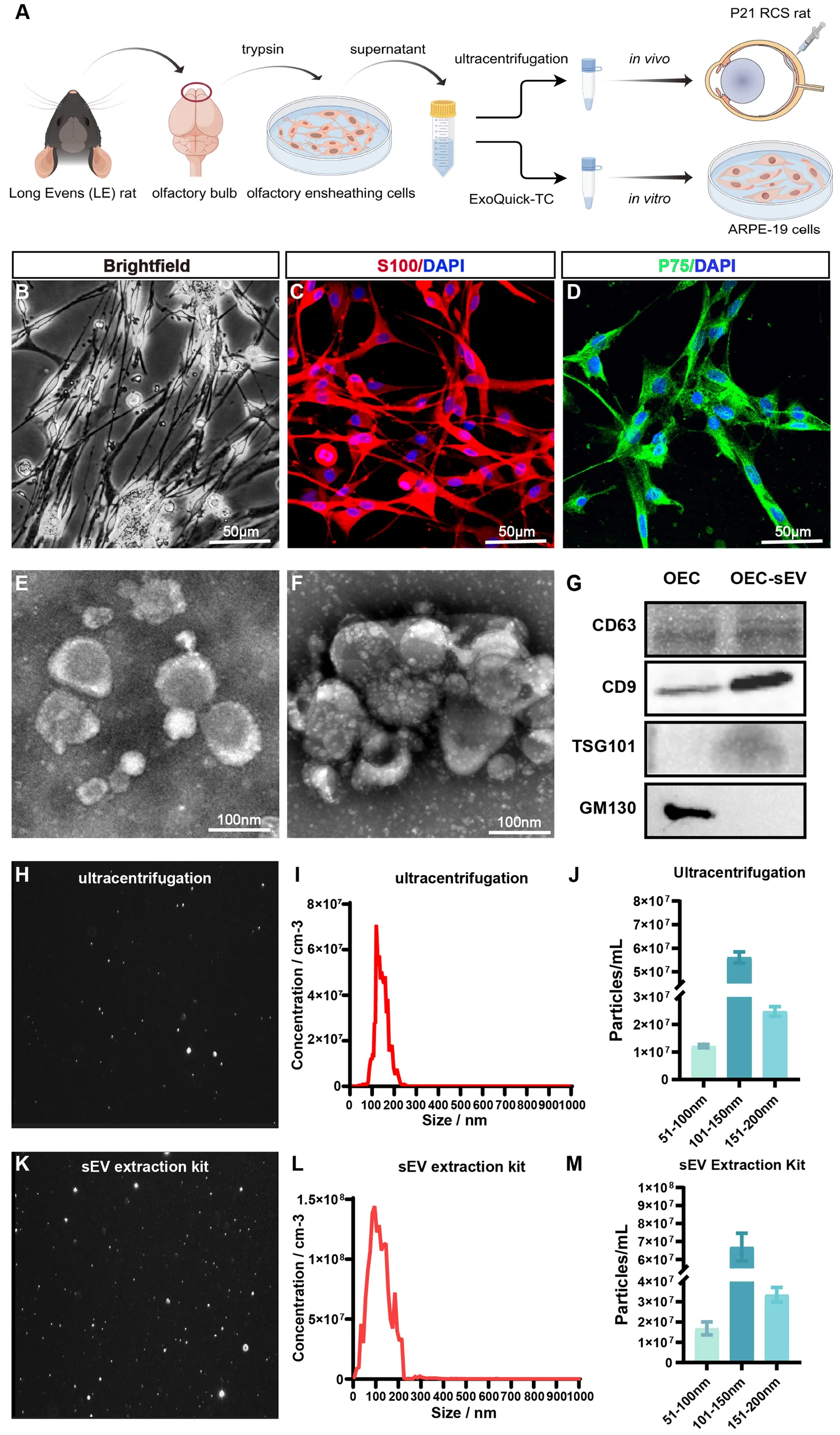
Characterization of OEC-derived sEVs. (A) Schematic of *in vivo* and *in vitro* experiments. Created on www.figdraw.com (Copyright Code: SASSR444de); (B) Representative light microscopy image showing the typical morphology of OECs. Scale bars = 50 μm; (C and D) Immunofluorescence staining of OEC-specific markers S100 (green), P75 (red), and DAPI (blue). Scale bars = 50 μm; (E and F) TEM images of sEVs isolated via ultracentrifugation (E) and ExoQuick-TC (F). Scale bars = 100 nm; (G) Western blot analysis confirms the expression of positive and negative markers for sEVs (CD63, CD9, TSG101, GM130) in both OECs and OEC-sEVs isolated by ultracentrifugation. *n* = 3 biologically independent OEC cultures and OEC-sEV preparations. The images shown are representative of three independent experiments with similar results; (H and K) Optical microscope showing the Brownian motion and dispersion of OEC-sEVs isolated by supercentrifugation (H) and ExoQuick-TC (K), respectively; (I and L) NTA of OEC-sEVs isolated by supercentrifugation (I) and ExoQuick-TC (L); (J and M) Peak analysis of the distribution of OEC-sEVs isolated by supercentrifugation (J) and ExoQuick-TC (M). Data represent OEC-EVs isolated from *n* = 4 independent biological preparations, with each sample measured in technical triplicate. Error bars represent the mean ± SD. Variation across preparations reflects biological heterogeneity inherent in EV production. DAPI: 4’,6-diamidino-2-phenylindole; EVs: extracellular vesicles; NTA: nanoparticle tracking analysis; OEC: olfactory ensheathing cell; OEC-sEVs: olfactory ensheathing cell-derived small extracellular vesicles; SD: standard deviation; sEVs: small extracellular vesicles; TEM: transmission electron microscopy; TSG101: tumor susceptibility gene 101.

### OEC-sEVs preserved the retinal functions of RCS rats

RCS rats were randomly divided into two groups: one received PBS injections (RCS-PBS group), and the other received OEC-sEV treatment (RCS-sEV group). We employed FERG to evaluate the retinal electrical function. The a-wave of the FERG reflects the light-sensing capability of the photoreceptor cells, whereas the b-wave represents the transmission process of light signals from the photoreceptor cells to the bipolar and ganglion cells. The amplitude and latency time of these waves provide insights into the intensity of retinal cell activity and the speed of response to light stimulation, respectively. FERGs showed that, compared with the RCS-PBS group, the RCS-sEV group exhibited consistently higher a-wave amplitudes after sEV injection at all examined time points (weeks 1, 2, and 4). Notably, the disparity between the RCS-sEV group and the LE group progressively increased over time, suggesting that while OEC-sEV treatment provided functional improvement relative to the diseased control, the therapeutic effect remained below normal physiological levels [Figure 2A-D]. Analysis of b-wave amplitudes revealed that the RCS-sEV group demonstrated significantly elevated responses compared with the RCS-PBS group at weeks 1 and 2 post-injection. However, by week 4, this difference between the two groups was no longer statistically significant [Figure 2E-G]. Consistently, the functional benefits in both the OEC-sEV and OEC-PBS groups diminished over the observed time course. To ascertain the precise localization of injected OEC-sEVs and their uptake by RPE cells, IF staining was performed. In the injection area, PKH26-labeled OEC-sEVs were traced to aggregate in the subretinal spaces (SRS) post-transplantation, and great differences in the ONL thickness between grafted and non-grafted areas in the same retinas of RCS rats were observed [Supplementary Figure 1A-I]. The ONL thickness of the retina was significantly preserved in the OEC-sEV-grafted area compared to that in the non-grafted area [Supplementary Figure 1J and K]. We further explored whether the implanted OEC-sEVs protected the photoreceptors and found that the OEC-sEVs decreased the fluorescence intensity of TUNEL in the RCS-sEV group compared with that in the age-matched RCS-PBS group at weeks 2 and 4 [Figure 2H (1-9)]. Furthermore, immunostaining of arrestin and protein kinase C alpha (PKCA) exhibited stronger fluorescence intensity of cones in the SRS and more stretched dendrites of bipolar cells in the inner nuclear layer (INL) in the RCS-sEV group than in the RCS-PBS group [Figure 2I (1-6) and J (1-6)]. The data indicated that in the RCS-sEV group, the number of TUNEL-positive cells decreased, while ONL thickness increased, compared to the RCS-PBS group [Figure 2K and L], implying that the photoreceptors were preserved with OEC-sEV treatment. Additionally, the OEC-sEV injection enhanced the fluorescence intensities of arrestin and PKCA [Figure 2M and N], consistent with the immunostaining images. We utilized OECs as a positive control group to further evaluate the therapeutic effects of OEC-sEVs. The a-wave and b-wave amplitudes at week 2 in the OEC-sEV group increased significantly compared to those in the PBS and OEC-Cell groups [Supplementary Figure 2A-C]. However, the OEC-cell group showed a longer-lasting therapeutic effect than the OEC-sEV group, whereas the therapeutic effect in the OEC-sEV group diminished over time [Supplementary Figure 2D-F]. Overall, it was demonstrated that OEC-sEVs could effectively protect retinal structures and preserve retinal functions, and the therapeutic effect of the OEC-sEV group was better than that of the OEC-cell group in the short term.

**Figure 2:**
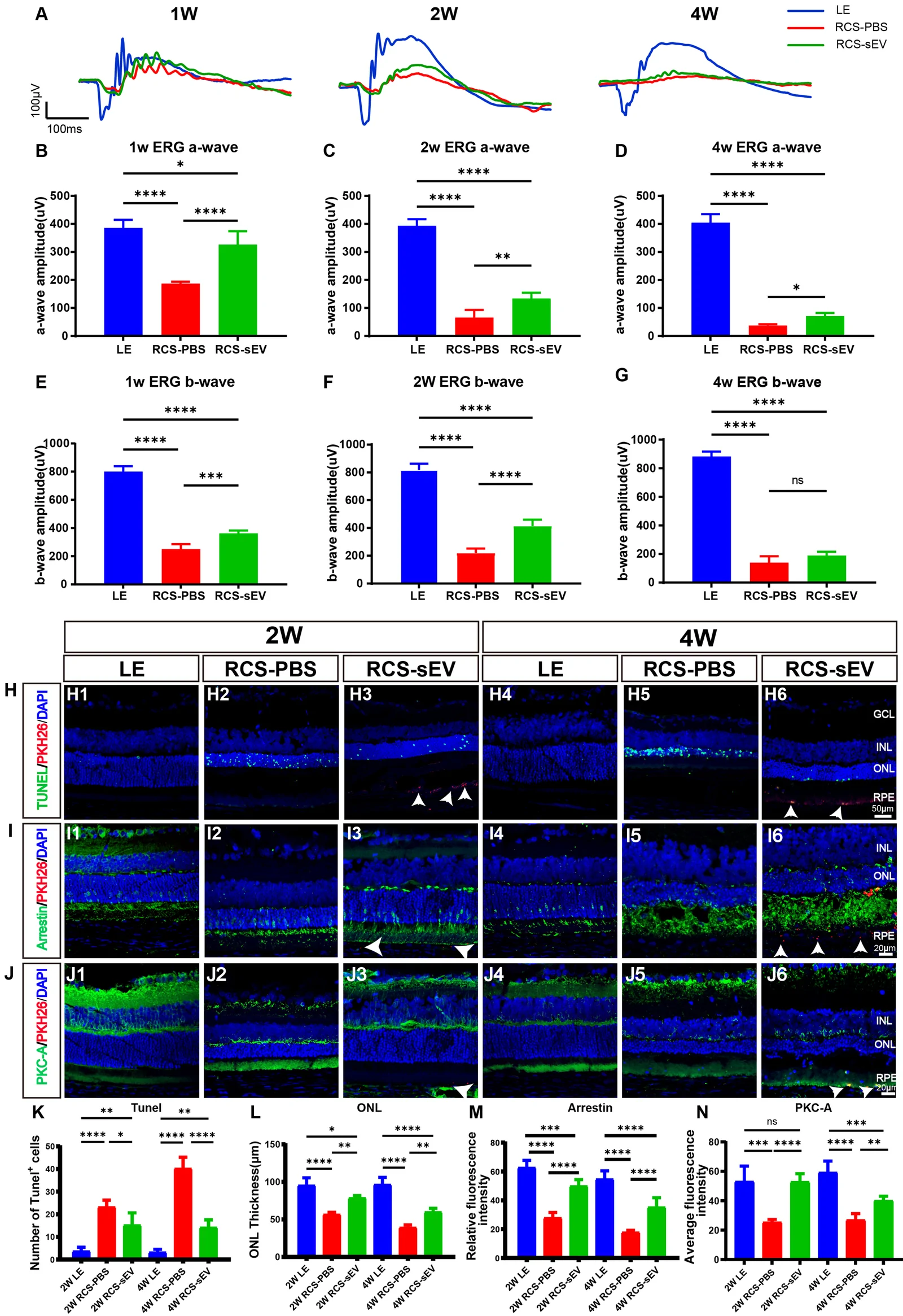
Protective effects of OEC-sEVs on photoreceptor and bipolar cell degeneration in RCS rats. (A) ERG graph of LE, RCS-PBS, and RCS-sEV groups at 1, 2 and 4 weeks post-injection; (B-D) Quantitative analysis of a-wave amplitude; (E-G) Quantitative analysis of b-wave amplitude; (H) PKH26-labeled OEC-sEV internalization (red) and TUNEL staining (green) in retinal sections of LE, RCS-PBS, and RCS-sEV groups at 2 and 4 weeks post-injection. Nuclei were stained with DAPI (blue). Scale bars = 50 μm; (I) PKH26-labeled OEC-sEVs internalization (red) and immunofluorescence staining of photoreceptors (Arrestin, green). Scale bars = 20 μm. (Since OEC-sEVs may fuse with the membrane or enter the intracellular space after uptake by the cell, PKH26 signaling may reflect the distribution or residue of sEV membrane components rather than the presence of intact sEVs.); (J) PKH26-labeled OEC-sEVs internalization (red) and immunofluorescence staining of bipolar cells (PKC-A, green). Scale bars = 20 μm; (K) Quantification of TUNEL-positive cells in LE, RCS-PBS, and RCS-sEV groups; (L) ONL thickness in LE retinas, PBS- and sEV-treated retinas; (M) Relative fluorescence intensity of Arrestin in LE, RCS-PBS, and RCS-sEV groups; (N) Relative fluorescence intensity of PKC-A in LE, RCS-PBS, and RCS-sEV groups. *n* = 4 biologically independent rats per group. Arrows show the presence of PKH26-labeled OEC-sEVs within the RPE layer. Data are represented as mean ± SD. ^*^P < 0.05, ^**^P < 0.01, ^***^P < 0.001, ^****^P < 0.0001, ns, no statistical difference. One-way ANOVA, followed by Tukey’s multiple comparisons test, was conducted for multiple comparisons. ANOVA: Analysis of variance; DAPI: 4’,6-diamidino-2-phenylindole; ERG: electroretinogram; GCL: ganglion cell layer; INL: inner nuclear layer; LE: Long Evans; OEC-sEVs: olfactory ensheathing cell-derived small extracellular vesicles; ONL: outer nuclear layer; PBS: phosphate-buffered saline; PKC-A: protein kinase C alpha; RCS: Royal College of Surgeons; RCS-PBS: Royal College of Surgeons rats treated with phosphate-buffered saline; RCS-sEV: Royal College of Surgeons rats treated with small extracellular vesicles; RPE: retinal pigment epithelium; SD: standard deviation; sEV: small extracellular vesicle; sEVs: small extracellular vesicles; TUNEL: terminal deoxynucleotidyl transferase dUTP nick end labeling.

### OEC-sEVs enrich phagocytosis-related proteins

To functionally characterize the OEC-sEVs, an in-depth proteomic analysis was performed. A total of 1,609 proteins were screened and identified as DEPs between OEC-sEVs and OECs. Among these, 487 DEPs were highly expressed in OEC-sEVs, and 1,122 DEPs exhibited higher expression levels in OECs [Figure 3A and B]. The heatmap of protein expression determined the homogeneity of the biological replicates in each group and the notable differences in each group [Figure 3C]. In KEGG enrichment analysis, a pathway related to “endocytosis” was identified as the most enriched pathway in OEC-sEVs. Furthermore, the genes targeted by OEC-sEVs were also associated with the pathway involved in “regulation of the actin cytoskeleton”, which plays a crucial role in phagocytosis [Figure 3D]. GO enrichment analysis of highly expressed DEPs in OEC-sEVs detected six enriched terms, including “endosomal transport”, “vacuolar transport” and “exocytosis” in the Biological Process (BP) [Figure 3E], “endosome membrane” and “early endosome” in the Cellular Component (CC) [Figure 3F], and “cell adhesion molecular binding” in the Molecular Function (MF) [Figure 3G], which implied the association of OEC-sEVs and phagocytosis. Given that the RCS rat model harbors a mutation in the *Mertk* gene, we specifically examined the expression profiles of MerTK and its associated phagocytic pathway components following OEC-sEV treatment. Proteins related to phagocytosis (MerTK and Gas6) and intracellular transport (Pros1, Rab5b, Rab7a and Rab5a), were expressed at higher levels in the OEC-sEVs group [Figure 4A]. Subsequently, to systematically investigate the functional relationships among DEPs associated with phagocytosis and the MerTK-related signaling pathway, we constructed a PPI network. Network analysis revealed interconnections between the phagocytic pathway-related DEPs and MerTK [Figure 4B]. To explore the biological implications of DEPs, we conducted GO and KEGG pathway enrichment analyses. GO analysis revealed the significant enrichment of multiple phagocytosis-associated terms. Within the BP category, the term “endosome transport via multivesicular body sorting pathway” was enriched [Figure 4C]. In the MF category, “integrin binding” and “cell adhesion molecule binding” emerged as key enriched terms [Figure 4D], whereas the CC category showed enrichment for “late endosome,” “endosome,” and “early endosome” [Figure 4E]. KEGG pathway analysis demonstrated significant enrichment of “endocytosis” and “PI3K-Akt signaling pathway” [Figure 4F], both of which are closely associated with phagocytic processes. Altogether, these data serve to identify candidate molecules and related pathways putatively associated with the observed phenotypic rescue. Accordingly, we hypothesized that OEC-sEVs might enhance RPE phagocytic activity in an RCS rat model via the MerTK-related signaling pathway.

**Figure 3:**
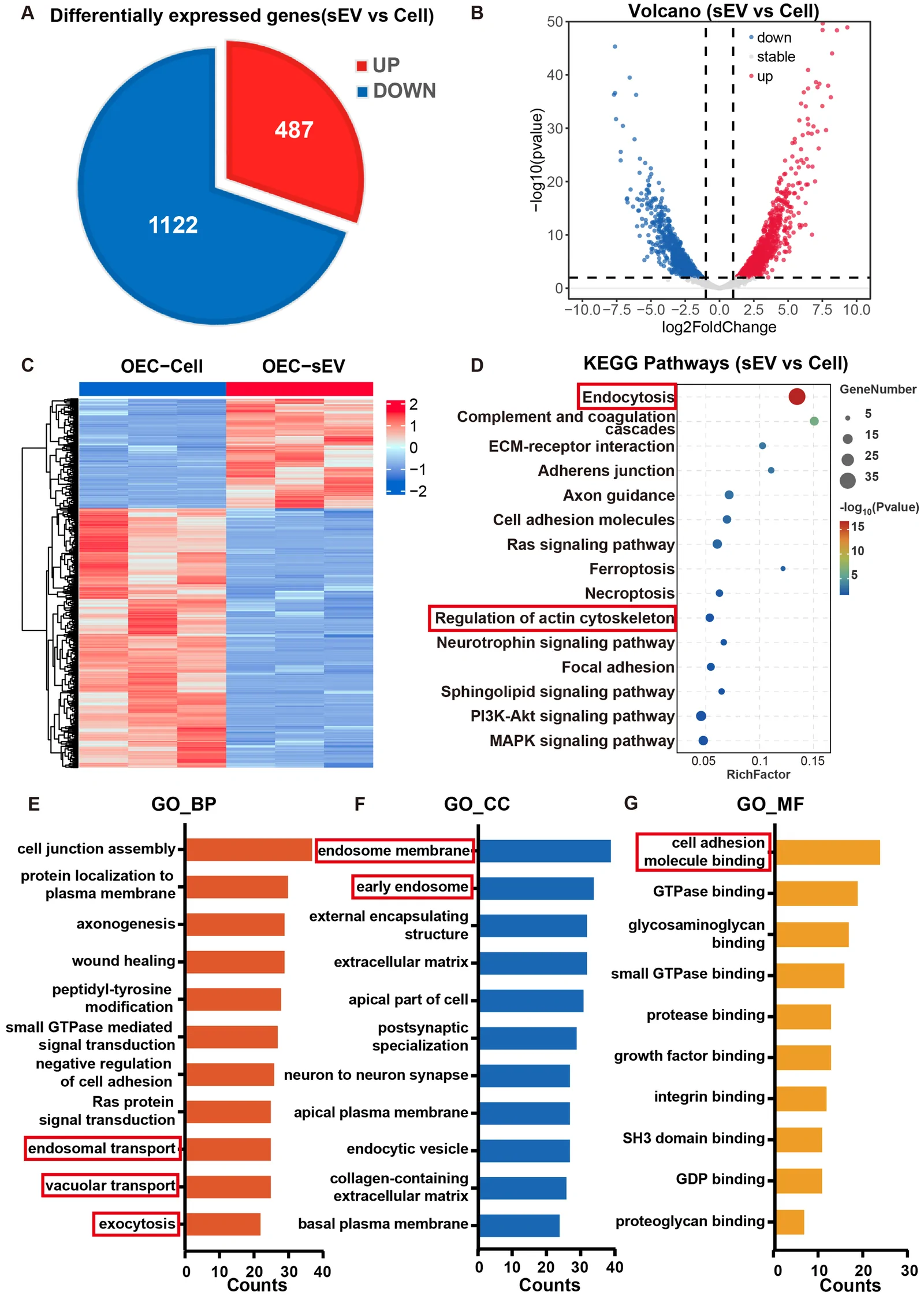
Proteomic profiling of OEC-sEVs *vs*. OEC whole-cell lysates. (A) Pie chart of differentially expressed proteins; (B) Volcano plot highlighting upregulated (red) and downregulated (blue) proteins in OEC-sEVs; (C) Heatmap of protein expression profiles; (D) KEGG analysis of highly expressed proteins in the OEC-sEV group; (E) GO (Biological Process) analysis of highly expressed proteins in the OEC-sEV group; (F) GO (Cellular Component) analysis of highly expressed proteins in the OEC-sEV group; (G) GO (Molecular Function) analysis of highly expressed proteins in the OEC-sEV group. *n* = 3 biologically independent OEC cultures and *n* = 3 biologically independent sEV preparations. Red boxes highlight the critical pathways associated with RPE phagocytosis. GO: Gene Ontology; KEGG: Kyoto Encyclopedia of Genes and Genomes; OEC: olfactory ensheathing cell; OEC-sEVs: olfactory ensheathing cell-derived small extracellular vesicles; RPE: retinal pigment epithelium; sEV: small extracellular vesicle; sEVs: small extracellular vesicles.

**Figure 4:**
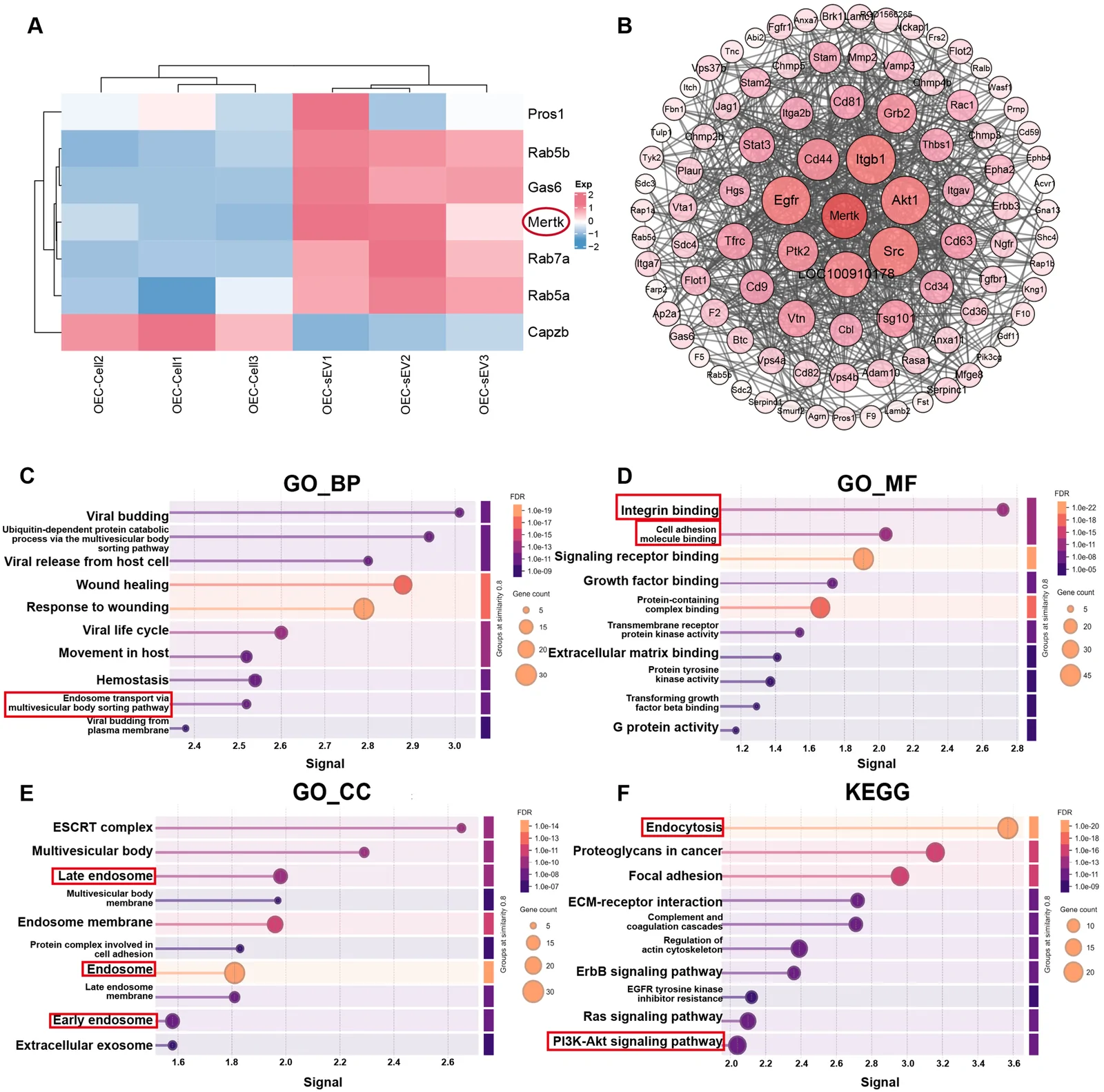
PPI network and functional analysis of OEC-sEVs. (A) Expression levels of phagocytosis-related proteins (Pros1, Rab5b, Gas6, MerTK, Rab7a, Rab5a) and Capzb in OEC-sEV groups *vs*. OEC groups; (B) PPI network analysis of the phagocytosis pathway-related DEPs and MerTK; (C) GO (Biological Process) analysis of highly expressed proteins related to phagocytosis in the OEC-sEV group; (D) GO (Molecular Function) analysis of highly expressed proteins related to phagocytosis in the OEC-sEV group; (E) GO (Cellular Component) analysis of highly expressed proteins related to phagocytosis in the OEC-sEV group; (F) KEGG pathway enrichment for highly expressed proteins related to phagocytosis in the OEC-sEV group. *n* = 3 biologically independent OEC cultures and *n* = 3 biologically independent sEV preparations. Red boxes highlight the critical pathways associated with RPE phagocytosis. Capzb: Capping actin protein of muscle Z-line subunit beta; DEPs: differentially expressed proteins; Gas6: growth arrest-specific protein 6; GO: Gene Ontology; KEGG: Kyoto Encyclopedia of Genes and Genomes; MerTK: MER proto-oncogene, tyrosine kinase; OEC: olfactory ensheathing cell; OEC-sEVs: olfactory ensheathing cell-derived small extracellular vesicles; PPI: protein-protein interaction; Pros1: protein S1; Rab5a: Ras-related protein Rab-5A; Rab5b: Ras-related protein Rab-5B; Rab7a: Ras-related protein Rab-7A; RPE: retinal pigment epithelium; sEV: small extracellular vesicle; sEVs: small extracellular vesicles.

### Injected OEC-sEVs were internalized by retinal pigment epithelial (RPE) cells and enhanced the expression of phagocytosis-related genes *in vivo*

To determine the significance of the visual cycle in the phagocytic function of RPE cells, we performed IF staining for RPE65, the pivotal functional protein integral to the visual cycle. PKH26-labeled OEC-sEVs were detected in specific SRS regions at 2 and 4 weeks. The density of OEC-sEVs changed, and they exhibited increased dispersion over time. Fewer OEC-sEVs were situated in the SRS at week 4 than at week 2, and OEC-sEVs were internalized into RPE cells at both weeks 2 and 4 [Figure 5A]. We further investigated the internalization of OEC-sEVs by phagocytic microglia. IF analysis revealed that a limited proportion of OEC-sEVs colocalized with Iba1-positive microglia, whereas the majority were phagocytosed by RPE cells [Supplementary Figure 3A and B]. Consequently, we propose that the protective effect of OEC-sEVs on the retina was primarily mediated by their interaction with RPE cells. Compared with the RCS-PBS group, the OEC-sEV group exhibited the continuity of the RPE layer and a higher fluorescence intensity level of RPE65, indicating that RPE was preserved by the injected OEC-sEVs [Figure 5B and C]. Collectively, these findings suggested that PKH26-labeled OEC-sEVs were specifically internalized by RPE cells rather than by microglia and exerted direct regulatory effects on RPE cells.

**Figure 5:**
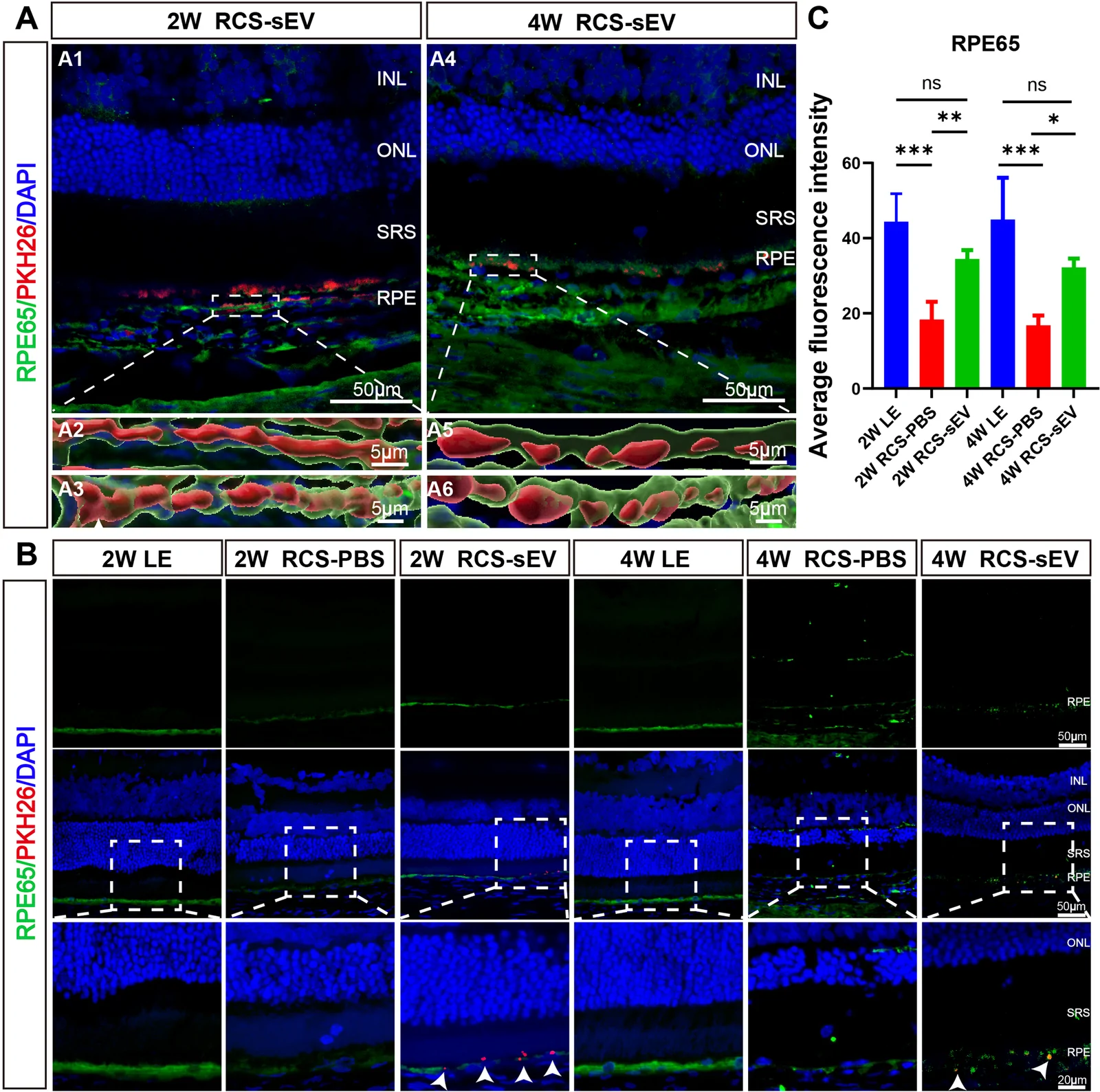
Internalization and phagocytic enhancement of RPE cells in RCS rats by OEC-sEVs. (A) Representative images showing the internalization of PKH26-labeled OEC-sEVs (red) in the RPE layer (RPE65, green) at 2 and 4 weeks post-injection; (A1, A4) Images of PKH26-labeled OEC-sEVs (red) and fluorescent staining of RPE65 (green) at 2 and 4 weeks post-injection. Scale bar = 50 μm; (A2, A3, A5, A6) 3D surface reconstruction images of OEC-sEVs and RPE cells rotated 0° (A2, A5) and 45° (A3, A6) in the horizontal plane. Scale bar = 5 μm; (B) Co-localization of PKH26-labeled OEC-sEVs (red) with RPE cells (RPE65, green) in the RCS-PBS and RCS-sEV groups at 2 and 4 weeks post-injection. Scale bar = 50 μm (original images); scale bar = 20 μm (enlarged images); (C) Quantitative analysis of RPE65 fluorescence intensity in the RCS-PBS and RCS-sEV groups at 2 and 4 weeks post-injection. The dashed boxes denote the regions selected for magnification in the subsequent panels. The arrows indicate the presence of PKH26-labeled OEC-sEVs within the RPE layer. *n* = 4 biologically independent rats per group. Data are represented as mean ± SD. ^*^P < 0.05, ^**^P < 0.01, ^***^P < 0.001, ns, no statistical difference. One-way ANOVA, followed by Tukey’s multiple comparisons test, was conducted for multiple comparisons. ANOVA: Analysis of variance; DAPI: 4’,6-diamidino-2-phenylindole; INL: inner nuclear layer; OEC-sEVs: olfactory ensheathing cell-derived small extracellular vesicles; ONL: outer nuclear layer; PBS: phosphate-buffered saline; RCS: Royal College of Surgeons; RCS-PBS: Royal College of Surgeons rats treated with phosphate-buffered saline; RCS-sEV: Royal College of Surgeons rats treated with small extracellular vesicles; RPE: retinal pigment epithelium; RPE65: retinal pigment epithelium-specific 65 kDa protein; SD: standard deviation; SRS: subretinal space.

POS phagocytosis by RPE cells is a complicated biochemical and physiological process. As reported, POS can be recognized, bound, and endocytosed by RPE cells via specific membrane receptors, including MerTK, αvβ5 integrin, etc. Following endocytosis, POS is directed towards degradation by the cellular machinery with the intracellular navigation of Rab GTPase family members^[58-60]^ [Figure 6A]. To determine whether OEC-sEVs improved the phagocytic function of RPE cells and the underlying mechanism, we analyzed the signaling pathways involved in phagocytosis. The qRT-PCR results showed that the administration of OEC-sEVs promoted the expression of *Mertk*, *Gas 6*, *Pros 1*, *Rab 5a,* and *Rab 7a* at week 2 post-injection [Figure 6B]. However, the promotional effect of OEC-sEVs declined at week 4 [Figure 6C]. Western blot analysis revealed that Mertk and Rab5a expression levels in RPE cells were upregulated by OEC-sEVs at week 2. At 4 weeks, MerTK protein was barely detectable, and the protein levels of Rab5a and Rab7a were comparable to those in the control group, with no significant differences detected [Figure 6D-G]. Additionally, western blot analysis was performed to examine the downstream signaling molecules of MerTK, namely PI3K and phosphorylated PI3K (p-PI3K). The p-PI3K/PI3K ratio was consistently higher in the RCS-sEV group than in the RCS-PBS group at weeks 2 and 4 [Figure 6H-K]. These findings indicate that OEC-sEVs upregulated MerTK and its downstream signaling molecule, PI3K. Collectively, these findings verified that OEC-sEVs may enhance the phagocytic activity of RPE cells through a MerTK-related pathway.

**Figure 6:**
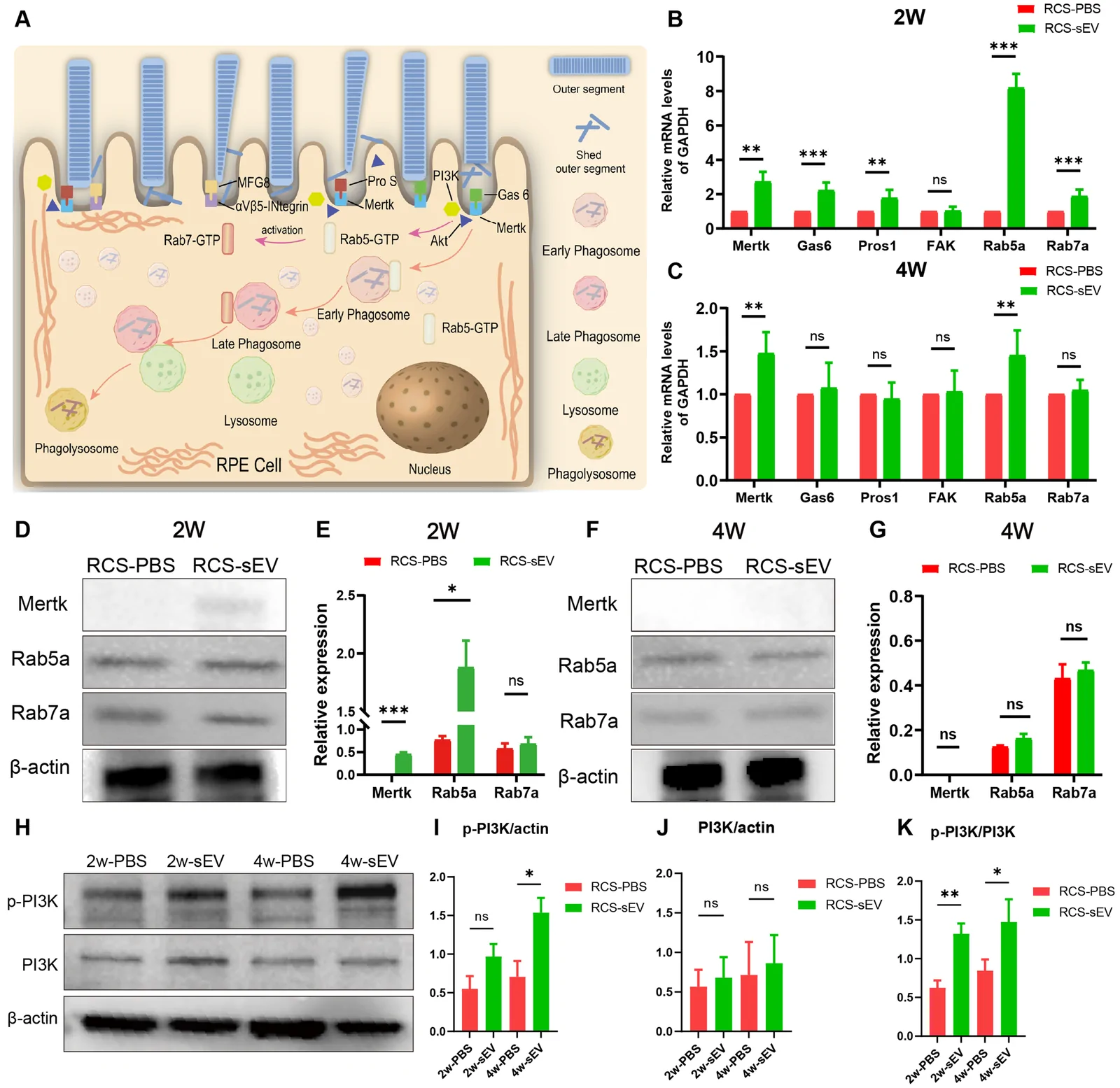
OEC-sEVs promote phagocytic function in RPE cells. (A) Schematic of POS phagocytosis by RPE cells. Inspired by Yang *et al*. (2021)^[61]^; (B) qRT-PCR analysis of MerTK, Gas6, Pros1, FAK, Rab5a, and Rab7a at week 2; (C) qRT-PCR analysis of MerTK, Gas6, Pros1, FAK, Rab5a, and Rab7a at week 4; (D and E) Western blot analysis of MerTK, Rab5a, Rab7a, and β-actin protein levels at week 2; (F and G) Western blot analysis of MerTK, Rab5a, Rab7a, and β-actin protein levels at week 4; (H-K) Western blot analysis of p-PI3K, PI3K, and β-actin protein levels at week 2 and week 4. *n* = 3 biologically independent rats per group. For each biological replicate, three technical replicates were performed. Data are represented as mean ± SD. ^*^P < 0.05, ^**^P < 0.01, ^***^P < 0.001, ns, no statistical difference. Student’s *t*-test was applied. β-actin: Beta-actin; FAK: focal adhesion kinase; Gas6: growth arrest-specific protein 6; GAPDH: glyceraldehyde-3-phosphate dehydrogenase; MerTK: MER proto-oncogene, tyrosine kinase; OEC-sEVs: olfactory ensheathing cell-derived small extracellular vesicles; PI3K: phosphoinositide 3-kinase; p-PI3K: phosphorylated phosphoinositide 3-kinase; POS: photoreceptor outer segments; Pros1: protein S1; qRT-PCR: quantitative real-time polymerase chain reaction; Rab5a: Ras-related protein Rab-5A; Rab7a: Ras-related protein Rab-7A; RPE: retinal pigment epithelium; SD: standard deviation.

### OEC-sEVs promoted POS phagocytosis by RPE cells and protected RPE cells and photoreceptors *in vivo*

Given that phototransduction is a fundamental function of photoreceptors^[62]^, it is essential to examine whether surviving photoreceptors retain their capacity for phototransduction at the molecular level. To this end, we performed IF staining for opsin, a key protein integral to rhodopsin that is pivotal in the phototransduction cascade. As depicted in Supplementary Figure 4A (A1, A3, A5, A7, A9) and (A11), there was almost no green fluorescence (opsin) near the RPE layer in RCS-PBS groups. However, the colocalization of the opsin (green fluorescence) and PKH26-labeled sEVs (red fluorescence) in the RPE layer was observed at week 2 [Supplementary Figure 4A (A2, A6, A10)] and at week 4 in the RCS-sEV group [Supplementary Figure 4A (A4, A8, A12)]. Furthermore, the fluorescence intensity and the number of opsin-positive cells increased after OEC-sEV treatment [Supplementary Figure 4B and C]. These observations supported that RPE phagocytosis was augmented after OEC-sEV transplantation.

The degeneration of RPE cells is characterized morphologically by irregular cell shapes, heterogeneous cell sizes, and disorganized cellular arrangements^[63]^. As degeneration progressed, the RPE cells in the RCS-PBS group displayed elongated or degenerated morphologies and variable cell sizes. In contrast, RPE cells in the LE and RCS-sEV groups demonstrated a more uniform cell size, regular hexagonal mosaic structure, and more orderly arrangement than those in the RCS-PBS group, especially at week 2. Moreover, IF staining revealed a greater degree of co-localization of green fluorescence (opsin) with RPE cells in the RCS-sEV group compared to that in the RCS-PBS group [Figure 7A]. The Feret diameter distribution and statistical analyses across the experimental groups showed that the LE, RCS-PBS, and RCS-sEV groups exhibited comparable mean Feret diameters and SDs at 2 days post-injection [Figure 7B-D], consistent with the early stage of retinal degeneration in RCS rats. However, at 2 weeks post-injection, both the RCS-PBS and RCS-sEV groups showed significantly higher mean Feret diameters and SDs than the LE group. Notably, the RCS-PBS group showed higher mean values and SDs than the RCS-sEV group [Figure 7E-G]. These findings indicate that disease progression in RCS rats was associated with increased RPE cell size and reduced size uniformity, whereas OEC-sEV treatment effectively ameliorated RPE cellular polymorphisms. Besides, the average size of RPE cells in the RCS-PBS group was significantly larger than that in the LE and RCS-sEV groups at week 2, consistent with the results of the Feret diameter analysis [Figure 7H]. In addition, the number of fluorescent particles (opsin) was significantly higher in the RCS-sEV and LE groups than in the RCS-PBS group at week 2, demonstrating that OEC-sEVs sustained the phagocytic function of RPE cells [Figure 7I]. These results indicated that OEC-sEVs rescued the degeneration of RPE cells and enhanced the phagocytic activity of RPE cells towards POS.

**Figure 7:**
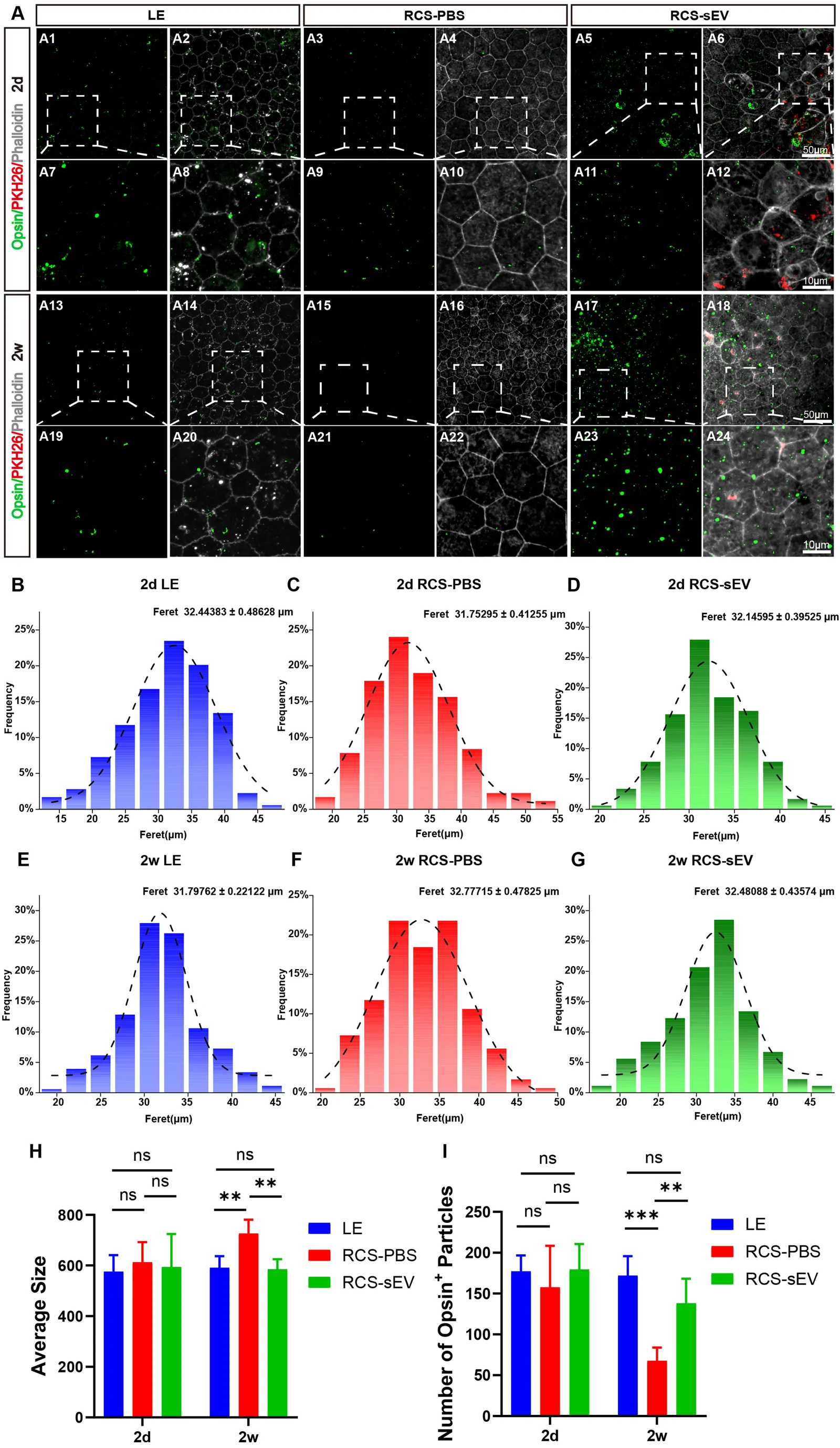
OEC-sEVs enhance RPE phagocytic activity in RCS rats. (A) Fluorescent staining of Phalloidin (grey) and Opsin (green) in retinal sections in LE, RCS-PBS, and RCS-sEV groups at day 2 (A1-A12) and week 2 (A13-A24). (A1-A6) Scale bar = 50 μm. (A7-A12) Scale bar = 10 μm. (A13-A18) Scale bar = 50 μm. (A19-A24) Scale bar = 10 μm. The dashed boxes denote the regions selected for magnification in the subsequent panels; (B-D) Histogram of Feret diameter distribution of RPE cells with Gaussian function fitting in LE, RCS-PBS, and RCS-sEV groups at day 2; (E-G) Histogram of Feret diameter distribution of RPE cells with Gaussian function fitting in LE, RCS-PBS, and RCS-sEV groups at week 2; (H) The average sizes of RPE cells in LE, RCS-PBS, and RCS-sEV groups at day 2 and week 2; (I) Quantitative analysis of the number of fluorescent particles per square at day 2 and week 2. *n* = 4 biologically independent rats per group. Data are represented as mean ± SD. ^**^P < 0.01, ^***^P < 0.001, ns, no statistical difference. One-way ANOVA, followed by Tukey’s multiple comparisons test, was conducted for multiple comparisons. ANOVA: Analysis of variance; LE: Long Evans; OEC-sEVs: olfactory ensheathing cell-derived small extracellular vesicles; PBS: phosphate-buffered saline; RCS: Royal College of Surgeons; RCS-PBS: Royal College of Surgeons rats treated with phosphate-buffered saline; RCS-sEV: Royal College of Surgeons rats treated with small extracellular vesicles; RPE: retinal pigment epithelium; SD: standard deviation.

### OEC-sEVs enhanced the phagocytosis and degradation functions of ARPE-19 cells *in vitro*

To further confirm whether the phagocytic function of RPE cells was promoted post-injection *in vitro*, we utilized pHrodo^TM^ BioParticles^TM^ conjugates to assess phagocytic activity. UNC2881, a specific Mer tyrosine kinase inhibitor, was employed to impair the phagocytic capacity of ARPE-19 cells. Western blot analysis demonstrated that phosphorylated MerTK (p-MerTK) relative to total MerTK expression was more effectively suppressed in the 50 µM UNC2881 group than in the 1, 3, 5, 10, and 25 µM UNC2881 groups [Supplementary Figure 5A-D]. Likewise, the density of the red phagocytosed particles was more obviously decreased in the 50 µM UNC2881 group than in the 5 µM UNC2881 group [Supplementary Figure 5E]. Thus, 50 µM UNC2881 was chosen for the subsequent experiments. As shown in Figure 8A, ARPE-19 cells engulfed a considerable number of fluorescent particles in the UNC2881-sEV group, even more prominently than in the control group. Flow cytometric analysis revealed that UNC2881 decreased the fluorescence intensity in ARPE-19 cells, which was significantly elevated only in the UNC2881-sEV group [Figures 8B-E]. It demonstrated that the 50 µM UNC2881 group exhibited lower fluorescence intensity than the 50 µM UNC2881-sEV group [Figure 8F]. We subsequently applied POS of porcine eyes to further verify the effect of OEC-sEVs on ARPE-19 cells in the following experiments. As expected, OEC-sEVs promoted the endocytosis of POS by ARPE-19 cells [Supplementary Figure 6A]. The fluorescence intensity of POS in ARPE-19 cells was considerably higher in the 50 µM UNC2881-sEV group than in the 50 µM UNC2881 group [Supplementary Figure 6B]. At 24 h post-injection, we detected β-hydroxybutyric acid, a decomposition resulting from the endocytosis of POS by ARPE-19 cells, in each group. As shown in Supplementary Figure 6C, compared with the control group, treatment with 50 µM UNC2881 decreased the level of β-hydroxybutyric acid, which was greatly increased after OEC-sEVs treatment. These results suggested that OEC-sEVs partially compensated for the phagocytic deficits in ARPE-19 cells *in vitro*. All full Western blot membrane images presented in this study are included in Supplementary Figure 7.

**Figure 8:**
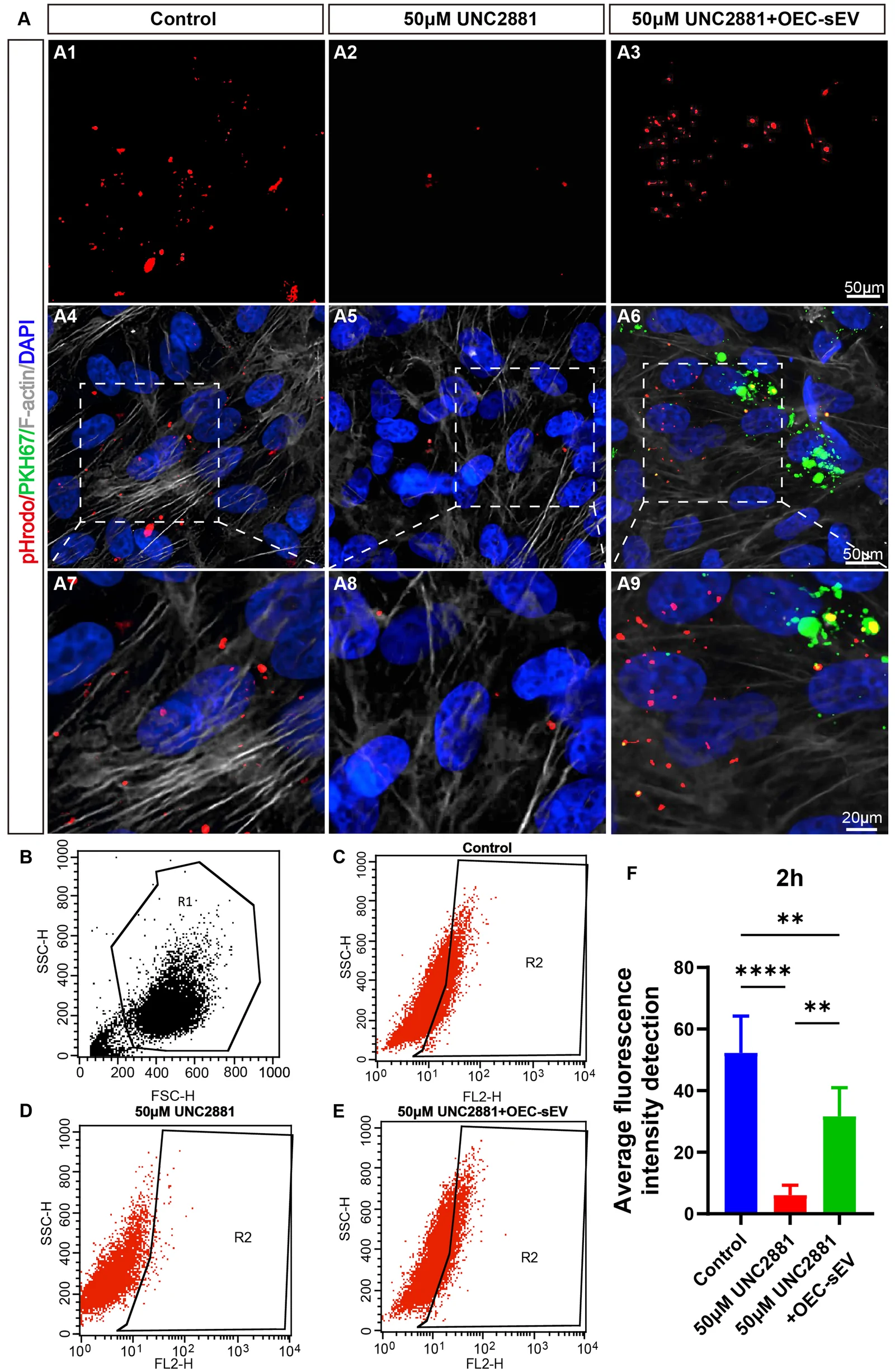
OEC-sEVs rescue UNC2881-induced phagocytic suppression in ARPE-19 cells. (A) Visualization of pHrodo^TM^ BioParticles^TM^ conjugates and PKH67, and fluorescent staining of F-actin in the control group, 50 μM UNC2881 group, and 50 μM UNC2881 + OEC-sEV group. Scale bar = 50 μm (original images); scale bar = 20 μm (enlarged images). The dashed boxes denote the regions selected for magnification in the subsequent panels; (B-E) Flow cytometry analysis of pHrodo^TM^ dyes in all three groups. R1 gate: Initial gating on FSC-H *vs*. SSC-H plot to identify the target cell population based on size and granularity, excluding debris and cell aggregates. R2 gate: pH-sensitive pHrodo^TM^ dye-positive population within the viable cell gate; (F) Average fluorescence intensity detection of pHrodo^TM^ dyes. *n* = 3 biologically independent cell cultures per group. Data are represented as mean ± SD. ^**^P < 0.01, ^****^P < 0.0001. One-way ANOVA, followed by Tukey’s multiple comparisons test, was conducted for multiple comparisons. ANOVA: Analysis of variance; ARPE-19: adult retinal pigment epithelial cell line-19; F-actin: filamentous actin; FSC-H: forward scatter height; OEC-sEVs: olfactory ensheathing cell-derived small extracellular vesicles; SD: standard deviation; SSC-H: side scatter height.

## DISCUSSION

Previous studies have reported that OEC-sEVs can serve as neuromodulators, exert neuroprotective effects, prevent neural apoptosis, and enhance neural regeneration^[36,64,65]^. However, whether OEC-sEVs can augment the phagocytic activity of phagocytosis-deficient cells remains unclear. In terms of the robust phagocytic capabilities of OECs, we speculated that OEC-sEVs may contain substances that can trigger the phagocytosis of RPE cells. Our study demonstrated that OEC-sEVs were primarily internalized by RPE cells and significantly facilitated the engulfment of POS by RPE cells via the MerTK-related pathway, both *in vivo* and *in vitro*, thereby preserving the retinal function of RCS rats. Proteomic analysis revealed a significant difference in protein composition between OEC-sEVs and their parental cells (OECs). This also provides evidence that OEC-sEVs are enriched in phagocytosis-related protein cargoes. In conclusion, OEC-sEVs represent a promising cell-free strategy for treating retinal degeneration.

RP is an inherited retinal degenerative disease involving a causative gene. In recent years, mutations in the receptor tyrosine kinase gene *Mertk* have been implicated in the pathogenesis of RP^[66]^, making *Mertk* a focal point of research on disease-causing genes. In 2000, *Mertk* was first recognized as an inevitable cause of human early-onset retinal degeneration in RPE cells of RCS rats^[67,68]^. Additionally, gene transfer of *Mertk* rescued RPE phagocytic defects in RCS rats, thereby reinforcing the significance of MerTK in RPE phagocytic function^[39]^. Besides, MerTK also plays an important role in regulating the number of POS attached to RPE cells^[69]^.

Based on these findings, we explored the phagocytic regulatory potential of OEC-sEVs in neurodegenerative diseases characterized by phagocytic impairment. As illustrated above, we showed that OEC-sEVs significantly upregulated the expression of phagocytosis-related genes (MerTK and PI3K) in RPE cells. These findings highlight the multifaceted effects of OEC-sEVs on RPE function, particularly by enhancing phagocytosis, a process vital for the maintenance of retinal homeostasis. Moreover, other critical pathways, such as those related to the actin cytoskeleton, also play a crucial role in phagocytosis and warrant more attention. Reportedly, POS uptake seems to start with the expansion of the apical RPE membrane near the tip of the POS^[70]^. This process of membrane expansion is associated with the recruitment of F-actin and the rebuilding of the F-actin cytoskeleton^[71]^. Live-cell imaging has revealed that F-actin protrudes from RPE cells into the surrounding area of the POS tip, where the POS tip becomes shed from the intact POS, indicating that F-actin may trigger the scission and phagocytosis of the POS tip^[72]^. Furthermore, actin is crucial for the motility and degradation of the phagosome after POS engulfment, thereby maintaining the stability of phagocytic function^[73,74]^.

It was reported that interferon-gamma-modified sEVs (IFNγ-sEVs) significantly inhibited activated microglia in RCS rats, thereby attenuating the retinal inflammatory response^[53]^. This effect is attributed to the enhancement of the immune properties of MSCs by IFNγ pretreatment, facilitating the phagocytosis and clearance of MSC-derived sEVs by microglia, the resident immune cells of the CNS. In a related study, sEVs derived from mouse neural progenitor cells (mNPC-sEVs) were specifically internalized by microglia in RCS rats, leading to the inhibition of inflammatory signaling pathways in microglia and consequently protecting photoreceptor cells from apoptosis^[51]^. Although both studies highlight the therapeutic effects of sEVs through microglial targeting, the underlying mechanisms differ. We hypothesized that the significant species differences between mouse-derived parental cells and the rat model might influence the phagocytosis of mNPC-sEVs by microglia in RCS rats. In contrast to the aforementioned findings, our study indicates that OEC-sEVs were minimally phagocytosed by microglia but were readily endocytosed by RPE cells. This might result from the fact that both the origin of the OEC and the RP animal model are derived from rats, which exhibit minimal interspecies variation. Consequently, OEC-sEVs demonstrated low immunogenicity in RCS rats, resulting in their preferential endocytosis by RPE cells, which are in close proximity to the subretinal space and are the most sensitive primordial cells to sEV exposure, rather than by microglial cells.

However, some limitations and understudied questions still remain. This study was limited to proteomic analyses of OEC-sEVs, which may provide insufficient evidence. To enhance our understanding, further sequencing analyses, such as microRNA sequencing, are necessary. Integrating proteomic and transcriptomic analyses enables measurement of gene expression at both the RNA and protein levels. This approach facilitates a comprehensive understanding of the differences and complementarities between transcriptomic and proteomic studies, providing a holistic view of gene expression and regulation across various stages. Such an integrated analysis is expected to yield novel insights that may not be obtained using traditional single-discipline histological methods. Furthermore, while our proteomic data revealed enrichment of MerTK-related effectors (e.g., Gas6, Pros1), we acknowledge that RPE phagocytosis involves complementary receptors such as αvβ5 integrin and CD36, which were not assessed in our study. The focus of the current study on MerTK reflects both the genetic lesion in the RCS model and our OEC-sEV functional cargo profile. Future studies are required to address multireceptor coordination. Additionally, the generalizability of our findings remains to be established across other RP models with distinct genetic backgrounds or pathophysiological mechanisms. Specifically, it remains unclear whether OEC-sEVs would confer comparable therapeutic benefits in non-*Mertk* -deficient RP models, such as those involving Rho, Pde6b, or EYS mutations, which represent the majority of human RP cases. Future studies are warranted to address these unanswered questions and deepen our understanding of the molecular mechanisms underlying the effects of OEC-sEVs on RPE phagocytosis.

Although PKH labeling was employed in this study for its established utility in *in vivo* sEV tracking, it has limitations for quantitative interpretation owing to potential dye aggregation and false-positive signals^[75]^. Alternatives, such as Cyanine 5 maleimide^[76]^, di-8-ANEPPS^[77]^, and CD63-GFP^[78,79]^, may provide complementary approaches to minimize dye-related artifacts. In addition, this study lacked direct rescue experiments for MerTK loss-of-function, which will be addressed in future studies. The ARPE-19 cell line, hindered by its inherent inability to express critical visual cycle enzymes (RPE65 and CRALBP) and its compromised epithelial barrier properties, serves only as a fundamentally restricted platform for investigating RPE biology^[80]^. It is biologically incapable of recreating disastrous downstream cascades, including massive ferroptosis, neuroimmune infiltration, and irreversible architectural collapse, which define the chronic genetic absence of MerTK in living RCS rat^[48,81]^. Meanwhile, conventional static cultures eliminate *in vivo* fluid shear stress and systemic clearance. This inevitably causes massive extracellular vesicle (EV) accumulation in the culture medium, artificially amplifying the pathological paracrine effects of vesicle signaling, which are substantially diluted *in vivo* by dynamic vascular clearance and metabolism^[82,83]^. Future work should shift to 3D organ-on-a-chip and live-imaging to dissect phagocytic dysfunction cascades and model retinal microenvironments to overcome the current limitations.

This study focused on the role of OEC-sEVs in RP, drawing the core conclusion that the successfully obtained OEC-sEVs were rich in phagocytosis-related proteins and could be internalized by RPE cells. In RCS rats, OEC-sEVs delayed photoreceptor degeneration and improved retinal function by repairing phagocytic defects caused by MerTK deficiency in RPE cells. This study could provide new candidate therapies and experimental evidence for RP, deepen our understanding of the paracrine regulatory mechanisms of OECs, and offer a research framework that can be referenced for sEV-based treatments for other ophthalmic degenerative diseases.

In conclusion, OEC-sEVs are abundant in proteins associated with phagocytosis, which may ameliorate the phagocytic dysfunction of the RPE observed in a rat model of RP via the MerTK-related signaling pathway.

## DECLARATIONS

### Availability of data and materials

All data supporting the findings of this study are included within the article and its Supplementary Materials. Additional raw data are available from the corresponding author upon request.

### AI and AI-assisted tools statement

During the preparation of this manuscript, the AI tool Grammarly (version 4.0, released 2024-02) was used solely for language editing. The tool did not influence the study design, data collection, analysis, interpretation, or the scientific content of the work. All authors take full responsibility for the accuracy, integrity, and final content of the manuscript.

### Copyright

© The Author(s) 2026.
