# Supplementary material for: Olfactory ensheathing cell-derived extracellular vesicles protect the degenerated retinal pigment epithelium by delivering phagocytosis-related proteins
Source: Extracell Vesicles Circ Nucl Acids. 2026 Jul 29;7(3):1189–216. doi: 10.20517/evcna.2026.50 (PMC13673258; doi:10.20517/evcna.2026.50)
Supplement: Supplementary file 1 [file evcna-7-3-1189-SupplementaryMaterials.pdf]

Supplementary Materials

Olfactory ensheathing cell-derived extracellular vesicles protect the degenerated retinal pigment epithelium by delivering phagocytosis-related proteins

Siyu Chen[1,2#], Xi Lin[1,2#], Zhe Cha[1,2#], Hui Gao[1,2,3], Jing Zhou[1,2], Shujia Huo[1,2], Cao Yang[1,2], Jiahui Kang[1,2], Lingyue Mo[1,2], Lijuan Yan[1,2], Yijian Li[1,2], Xinying Hu[4], Luodan A[1,2], Xiaotang Fan[5], Jing Xie[1,2], Haiwei Xu[1,2]

[1]Department of Ophthalmology, The First Affiliated Hospital of Army Medical University (Southwest Hospital), Chongqing 400038, China.
[2]Chongqing Key Laboratory of Visual Injury and Regeneration, Chongqing 400038, China.
[3]Shigatse Branch of Xinqiao Hospital, 953th Hospital, Army Medical University (Third Military Medical University), Shigatse 857000, Xizang, China.
[4]Department of Ophthalmology, Jiangyin People's Hospital, Medical School of Southeast University, Jiangyin 214432, Jiangsu, China.
[5]Department of Military Cognitive Psychology, School of Psychology, Third Military Medical University (Army Medical University), Chongqing 400038, China.
[#]These authors contributed equally to this work.

Correspondence to: Prof. Haiwei Xu, Prof. Jing Xie, Department of Ophthalmology, The First Affiliated Hospital of Army Medical University (Southwest Hospital), Chongqing 400038, China. E-mail: xuhaiwei@tmmu.edu.cn; xiejingrain@tmmu.edu.cn; Prof. Xiaotang Fan, Department of Military Cognitive Psychology, School of Psychology, Third Military Medical University (Army Medical University), Chongqing 400038, China. E-mail: fanxiaotang2005@163.com

Supplementary Table 1. Antibody information

Antibody	Company	Product Number	Species	Dilution
S100	Beyotime	AF1945	Rabbit	1:150
P75	Beyotime	AF1033	Rabbit	1:150
PKCa	Abcam	ab32376	Mouse	1:500
RPE65	Santa Cruz	sc-390787	Mouse	1:100
F-actin	Invitrogen	MA1-80729	Mouse	1:1000
Arresttin	Sigma-Aldrich	ab15282	Rabbit	1:500
CD63	Invitrogen	MA5-35208	Rabbit	1:200
CD9	Invitrogen	MA5-31980	Rabbit	1:200
TSG101	bioworld	BS6042	Rabbit	1:1000
GM130	Boster	M05865-2	Mouse	1:1000
Opsin	Thermo Fisher	PA1-9517	Chicken	1:200
Tunel	Roche	12156792910	/	1:300
647 Phalloidin	Abcam	AB176759	/	1:2000
goat-rabbit 488	Invitrogen	A11008	/	1:500
goat-mouse 488	Invitrogen	A11011	/	1:500
goat-chicken 488	Invitrogen	A11039	/	1:500
goat-mouse 647	Invitrogen	A21235	/	1:500
goat-rabbit 568	Invitrogen	A11036	/	1:500
goat-mouse 568	Invitrogen	A11031	/	1:500

Supplementary Table 2. Primer sequences

primers	species	Forward 5’-3’	Reverse 5’-3’
Rab5a	Rat	CTTCAAAGGCAAGCAAGTCC	GCCTGGATTTGTGGTTCGT
Rab7a	Rat	GGAGGTGATGGTGGATGACA	TGGGGGCAGTCACATCAAAC
FAK	Rat	CCAAGTGCAGACCATCCAGT	AGGTCAGCCATGTTCTCTGC
Mertk	Rat	CGCTCAGACAATGGGTCGTA	CCAGAAGATGTTGACGGGCT
Gas6	Rat	GCCAGGTCTGCCATAACAAA	AGTGTACCCCTTGTCGCAGA
PROS1	Rat	AGCTAAGGAGGCCGTGATGA	TGCACTTTCCACCTTCCGAG
β-Actin	Rat	ACAACCTTCTTGCAGCTCCTC	CTGACCCATACCCACCATCAC

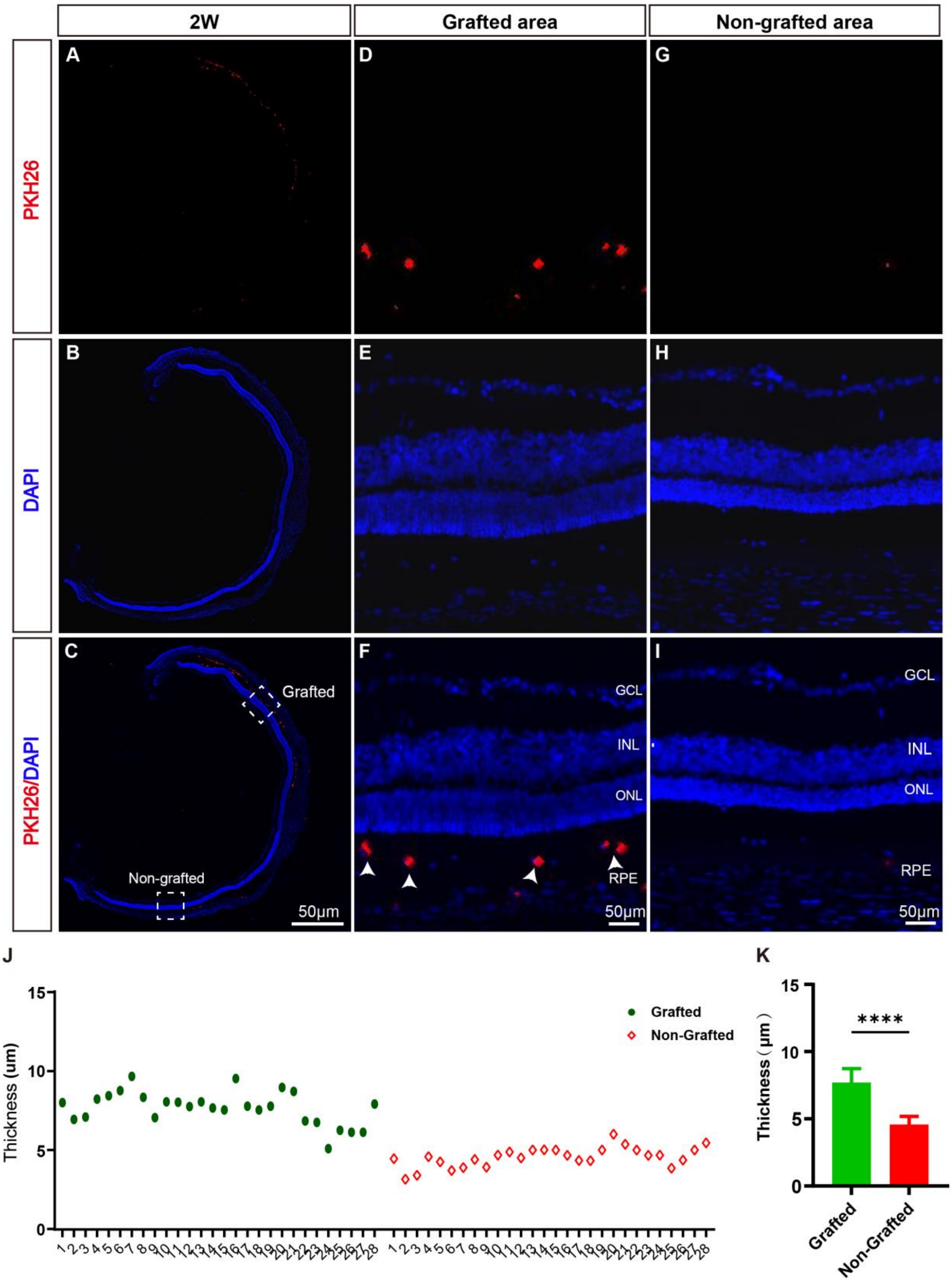

Supplementary Figure 1. Distribution areas and protective effects of OEC-sEVs on outer nuclear layer (ONL) post injection. (A-I) Comparison of PHK26-labeled OEC-sEVs grafted area and non-grafted area. Scale bar = 50 μm; (J-K) Comparison of thickness of ONL in Grafted group and Non-Grafted groups. Arrow shows the presence of PKH26-labeled OEC-sEVs within RPE layer. $n = 4$ biologically independent rats per group. Data are represented as mean ± SD. ****$P < 0.0001$. Student's t-test was applied.

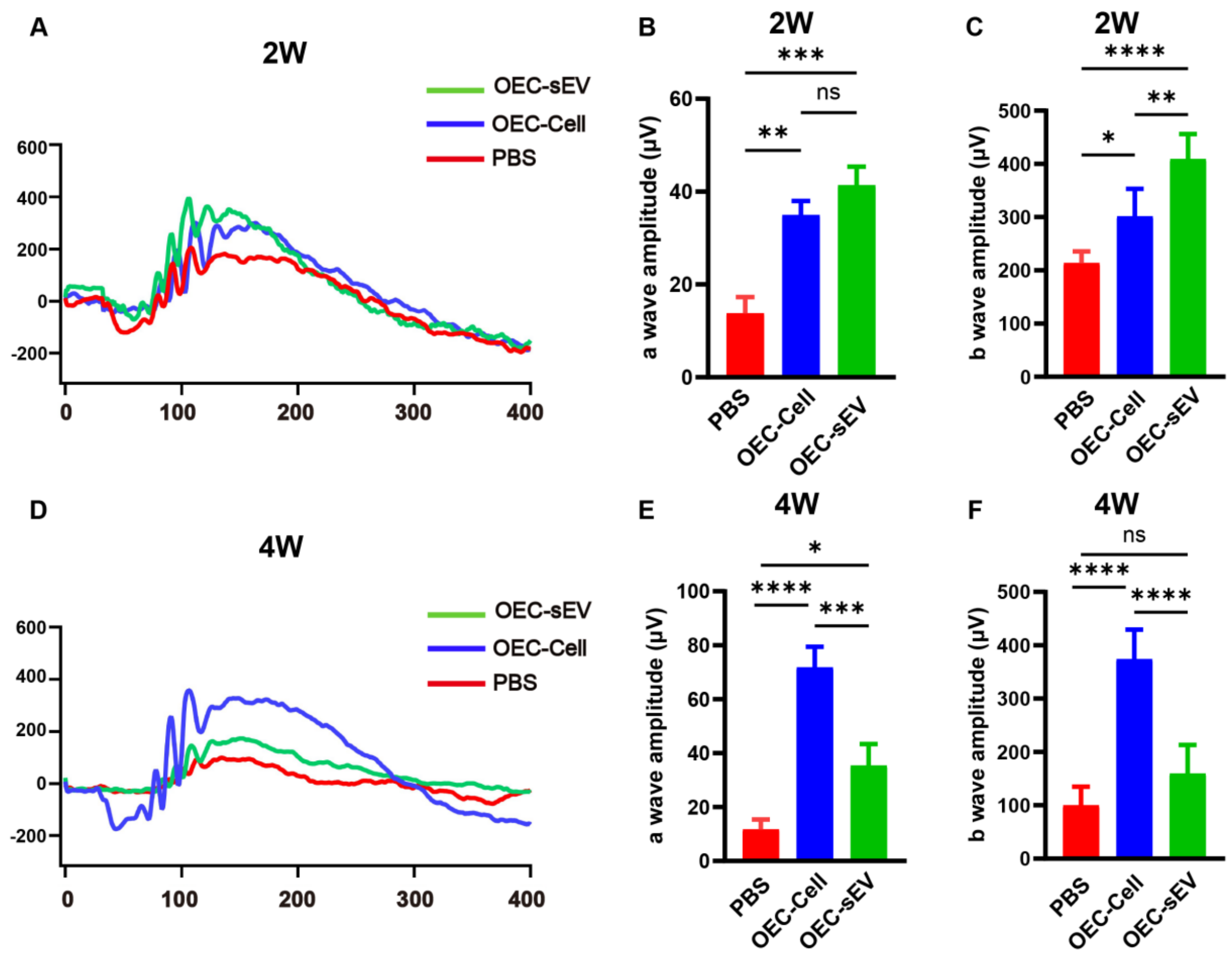

Supplementary Figure 2. Comparative neuroprotective effects of OEC-sEVs and OECs in RCS rats. (A and D) Representative ERG graphs at 2 and 4 weeks post-injection. (B, C, E, and F) Quantitative analysis of a-wave and b-wave amplitudes. n = 4 biologically independent rats per group. Data are represented as mean ± SD. *$P < 0.05$, **$P < 0.01$, ***$P < 0.001$, ****$P < 0.0001$, ns, no statistical difference. One-way ANOVA, followed by Tukey's multiple comparisons test, was conducted for multiple comparisons.

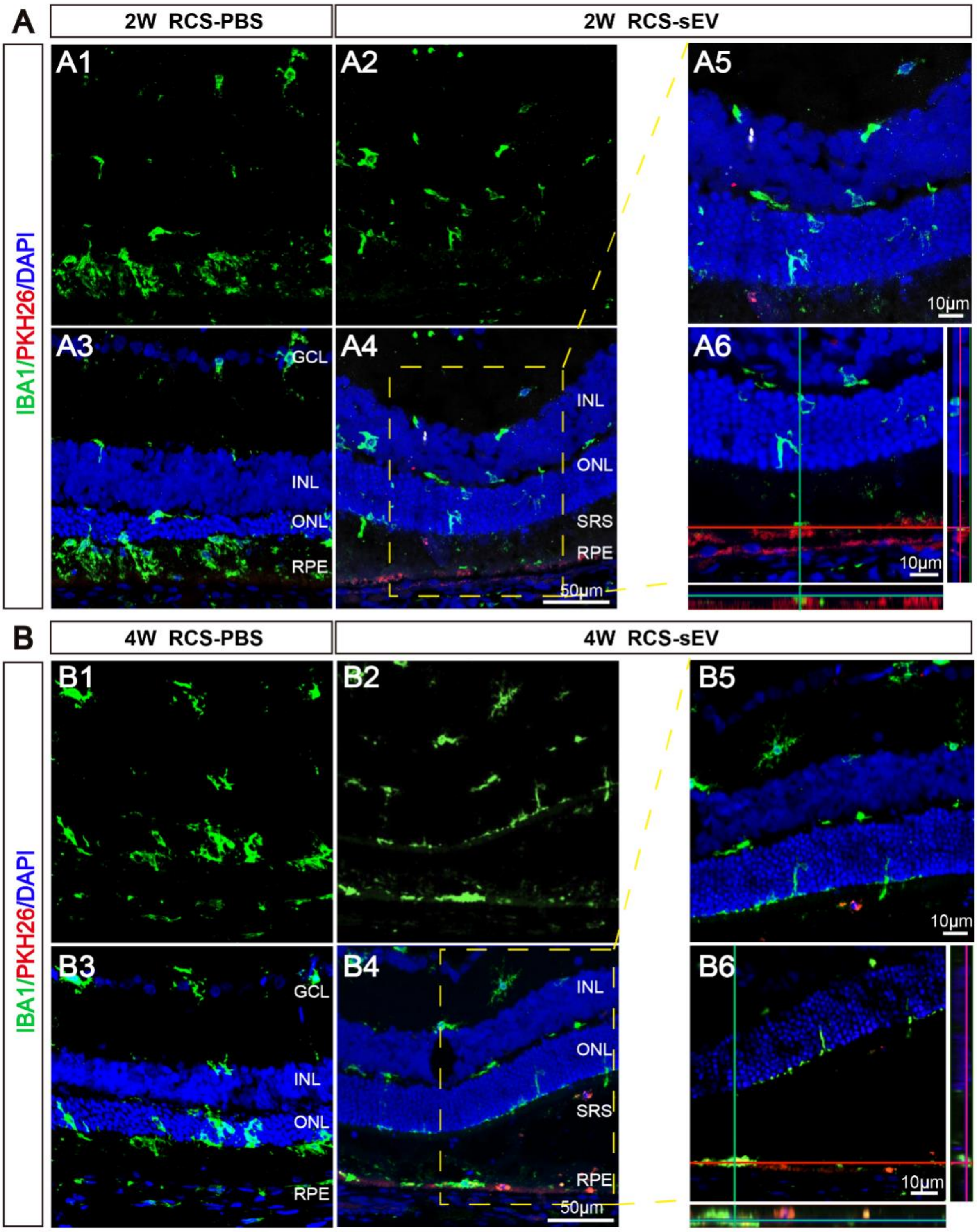

Supplementary Figure 3. The phagocytic uptake of OEC-sEVs by microglial cells. (A) Distribution of PKH26-labeled OEC-sEVs (red) within the retina and their co-localization with Iba1-positive microglia (green) at week 2. (A1-A4) Scale bar = 50 μm. (A5 and A6) Scale bar = 10 μm; (B) Distribution of PKH26-labeled OEC-sEVs (red) within the retina and their co-localization with Iba1-positive microglia (green) at week 4. (B1-B4) Scale bar = 50 μm. (B5 and B6) Scale bar = 10 μm.

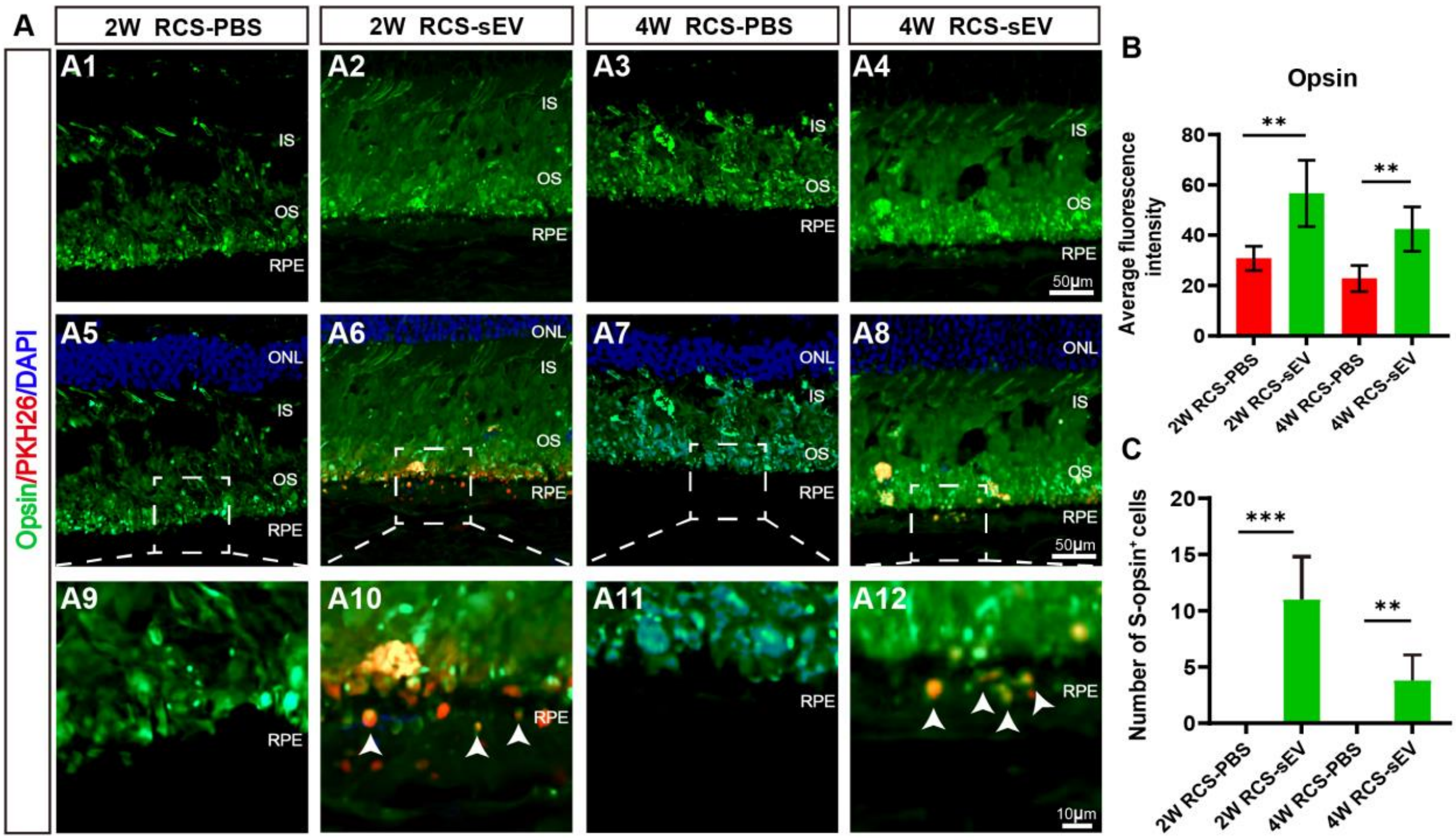

Supplementary Figure 4. Co-localization of OEC-sEVs with photoreceptors in the retinal pigment epithelial (RPE) layer of RCS rats. (A) PKH26-labeled OEC-sEVs (red) and Immunofluorescence staining of S-opsin (green) and DAPI (blue) at week 2 and week 4. (A1-A8) Scale bar = 50 μm. (A9-A12) Scale bar = 10 μm; (B) Average fluorescence intensity analysis of S-opsin in RCS-PBS and RCS-sEV groups at week 2 and 4; (C) Quantitative analysis of number of S-opsin positive cells within RPE layer in different groups. Arrow shows the co-localization of PKH26-labeled OEC-sEVs and S-opsin within RPE layer. n = 4 biologically independent rats per group. Data are represented as mean ± SD. $^{**}P < 0.01$, $^{***}P < 0.001$. Student's t-test was applied.

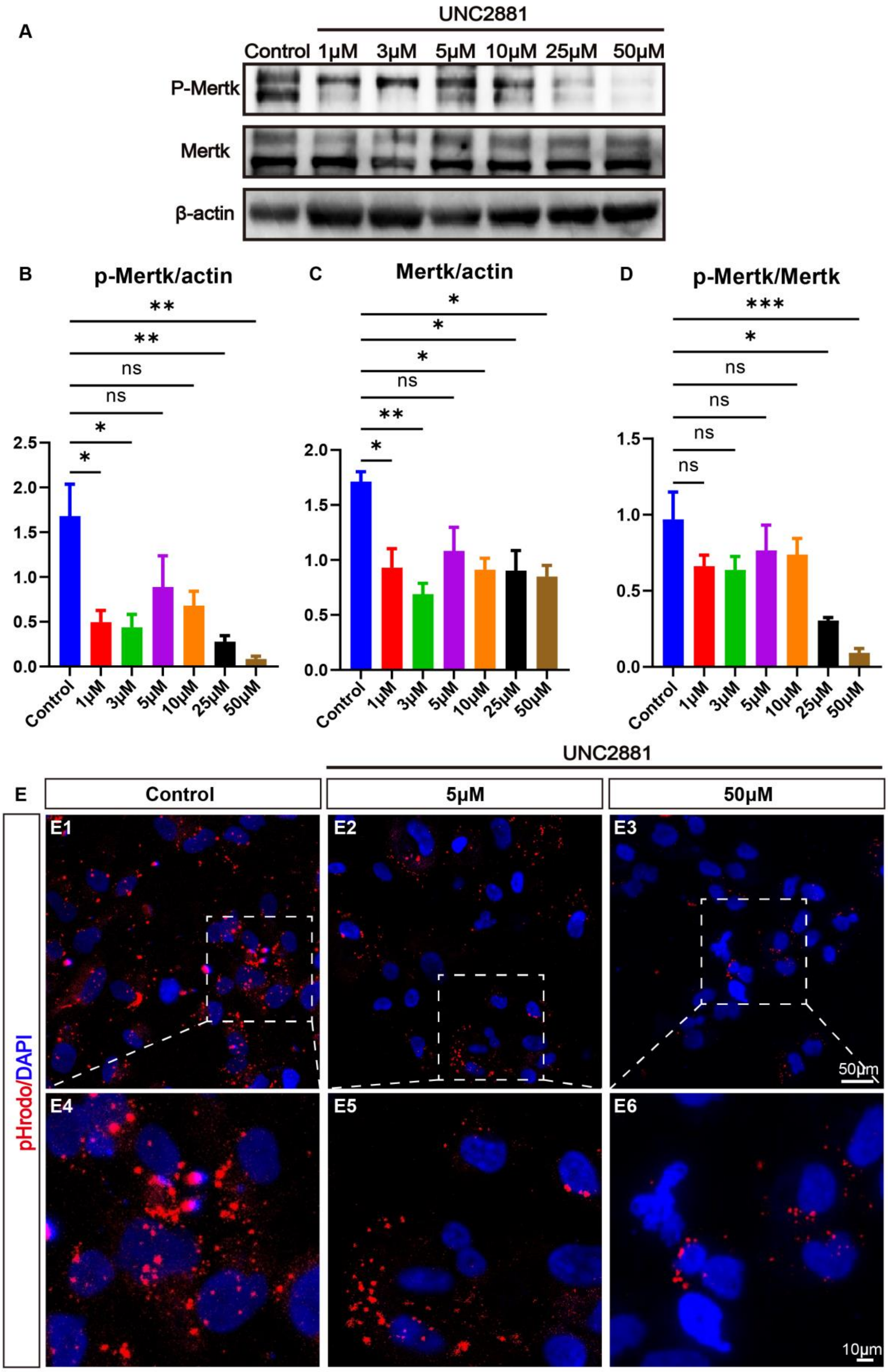

Supplementary Figure 5. Concentration gradient exploration of specific Mer tyrosine kinase inhibitor UNC2881. (A-D) Western blot analysis of p-Mertk and Mertk protein levels in ARPE-19 cells treated with UNC2881 (0, 1, 3, 5, 10, 25, and 50 μM); (E) Suppression effect of UNC2881 on phagocytic

activity of ARPE-19 cells. (E1 and E4) Control group without UNC2881 treatment. (E2 and E5) ARPE-19 cells treated with 5 μM UNC2881. (E3 and E6) ARPE-19 cells treated with 50 μM UNC2881. (E1-E3) Scale bar = 50 μm. (E4-E6) Scale bar = 10 μm. *n* = 3 biologically independent cell cultures per group. For each biological replicate, three technical replicates were performed. Data are represented as mean ± SD. *$P < 0.05$, **$P < 0.01$, ***$P < 0.001$, ns, no statistical difference. Student's *t*-test was applied.

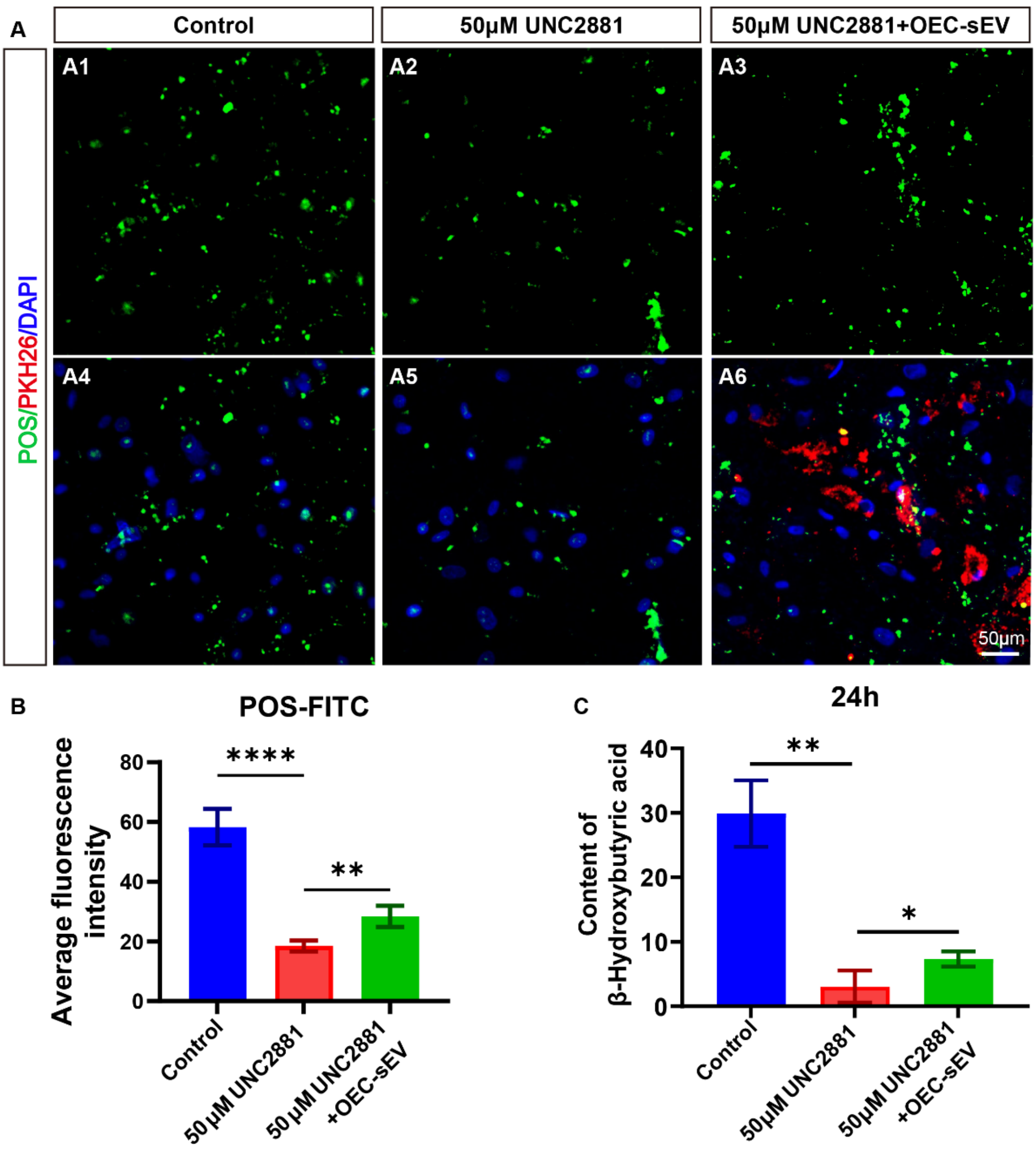

Supplementary Figure 6. OEC-sEVs enhance phagocytosis of POS in ARPE-19 cells under UNC2881 suppression. (A) Internalization of PKH26-labeled OEC-sEVs in rat retina and FITC-labeled POS in different groups. Scale bar = 50 μm; (B) Quantification of FITC-labeled POS fluorescence intensity across groups; (C) β-hydroxybutyric acid levels in all three groups after 24 h of treatment. *n* = 3 biologically independent cell cultures per group. Data are represented as mean ± SD. *$P < 0.05$, **$P < 0.01$, ****$P < 0.0001$. Student's *t*-test was applied.

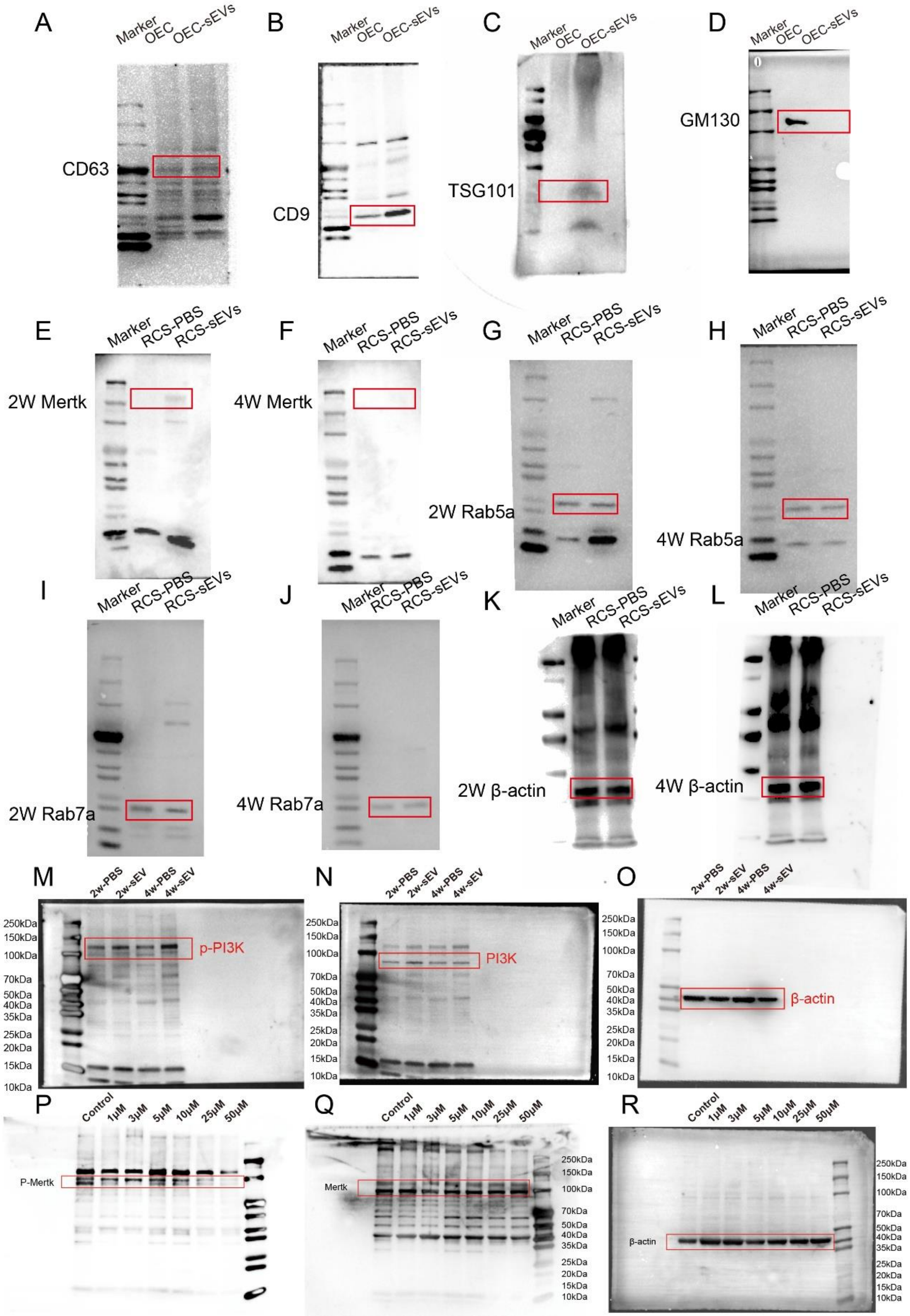
A
Marker
OEC
OEC-sEVs
CD63
B
Marker
OEC
OEC-sEVs
CD9
C
Marker
OEC
OEC-sEVs
TSG101
D
Marker
OEC
OEC-sEVs
GM130
E
Marker
RCS-PBS
RCS-sEVs
2W Mertk
F
Marker
RCS-PBS
RCS-sEVs
4W Mertk
G
Marker
RCS-PBS
RCS-sEVs
2W Rab5a
H
Marker
RCS-PBS
RCS-sEVs
4W Rab5a
I
Marker
RCS-PBS
RCS-sEVs
2W Rab7a
J
Marker
RCS-PBS
RCS-sEVs
4W Rab7a
K
Marker
RCS-PBS
RCS-sEVs
2W β-actin
L
Marker
RCS-PBS
RCS-sEVs
4W β-actin
M
2w-PBS
2w-sEV
4w-PBS
4w-sEV
250kDa
150kDa
100kDa
70kDa
50kDa
40kDa
35kDa
25kDa
20kDa
15kDa
10kDa
p-PI3K
N
2w-PBS
2w-sEV
4w-PBS
4w-sEV
250kDa
150kDa
100kDa
70kDa
50kDa
40kDa
35kDa
25kDa
20kDa
15kDa
10kDa
PI3K
O
2w-PBS
2w-sEV
4w-PBS
4w-sEV
250kDa
150kDa
100kDa
70kDa
50kDa
40kDa
35kDa
25kDa
20kDa
15kDa
10kDa
β-actin
P
Control
1μM
3μM
5μM
10μM
25μM
50μM
P-Mertk
Q
Control
1μM
3μM
5μM
10μM
25μM
50μM
Mertk
250kDa
150kDa
100kDa
70kDa
50kDa
40kDa
35kDa
25kDa
20kDa
15kDa
10kDa
R
Control
1μM
3μM
5μM
10μM
25μM
50μM
β-actin
250kDa
150kDa
100kDa
70kDa
50kDa
40kDa
35kDa
25kDa
20kDa
15kDa
10kDa

Supplementary Figure 7. Full Western blot membrane. The uncropped Western blot membrane corresponding to CD63 (A, related to Figure 1G), CD9 (B, related to Figure 1G), TSG101 (C, related to Figure 1G), GM130 (D, related to Figure 1G), 2w Mertk (E, related to Figure 6D), 4w Mertk (F, related to Figure 6F), 2w Rab5a (G, related to Figure 6D), 4w Rab5a (H, related to Figure 6F), 2w Rab7a (I, related to Figure 6D), 4w Rab7a (J, related to Figure 6F), 2w β-actin (K, related to Figure 6D) and 4w β-actin (L, related to Figure 6F), p-PI3K (M, related to Figure 6H), PI3K (N, related to Figure 6H), β-actin (O, related to Figure 6H), p-Mertk (P, related to Supplementary Figure 5A), Mertk (Q, related to Supplementary Figure 5A), β-actin (R, related to Supplementary Figure 5A).
